# Alterations in the Colonic Microbiota in Response to Osmotic Diarrhea

**DOI:** 10.1371/journal.pone.0055817

**Published:** 2013-02-08

**Authors:** Gregor Gorkiewicz, Gerhard G. Thallinger, Slave Trajanoski, Stefan Lackner, Gernot Stocker, Thomas Hinterleitner, Christian Gülly, Christoph Högenauer

**Affiliations:** 1 Institute of Pathology, Medical University of Graz, Graz, Austria; 2 Institute for Genomics and Bioinformatics, University of Technology, Graz, Austria; 3 Center for Medical Research, Medical University of Graz, Graz, Austria; 4 Division of Gastroenterology and Hepatology, Department of Internal Medicine, Medical University of Graz, Graz, Austria; Institute for Genome Sciences, University of Maryland School of Medicine, United States of America

## Abstract

**Background & Aims:**

Diseases of the human gastrointestinal (GI) tract are often accompanied by diarrhea with profound alterations in the GI microbiota termed dysbiosis. Whether dysbiosis is due to the disease itself or to the accompanying diarrhea remains elusive. With this study we characterized the net effects of osmotic diarrhea on the composition of the GI microbiota in the absence of disease.

**Methods:**

We induced osmotic diarrhea in four healthy adults by oral administration of polyethylene glycol 4000 (PEG). Stool as well as mucosa specimens were collected before, during and after diarrhea and 16S rDNA-based microbial community profiling was used to assess the microbial community structure.

**Results:**

Stool and mucosal microbiotas were strikingly different, with *Firmicutes* dominating the mucosa and *Bacteroidetes* the stools. Osmotic diarrhea decreased phylotype richness and showed a strong tendency to equalize the otherwise individualized microbiotas on the mucosa. Moreover, diarrhea led to significant relative shifts in the phyla *Bacteroidetes* and *Firmicutes* and to a relative increase in the abundance of *Proteobacteria* on the mucosa, a phenomenon also noted in several inflammatory and diarrheal GI diseases.

**Conclusions:**

Changes in microbial community structure induced by osmotic diarrhea are profound and show similarities to changes observed in other GI diseases including IBD. These effects so must be considered when specimens from diarrheal diseases (i.e. obtained by stratification of samples according to diarrheal status) or conditions wherein bowel preparations like PEG (i.e. specimens obtained during endoscopy) are used.

## Introduction

The human GI tract is populated by a complex community of microorganisms that play a pivotal role in the maintenance of health and the development of disease [1], [2]. Current knowledge indicates a crucial role for the GI microbiota in extracting nutrients from the diet, thereby influencing host metabolism, body growth and weight [3]. Moreover, it is a barrier against colonization with pathogens and is essential for mucosal homeostasis and for the maturation and correct function of the GI immune system [4]. Because our GI tract and its microbiota are interdependent, disease will affect both. A variety GI diseases including chronic inflammatory bowel disease (IBD), irritable bowel syndrome (IBS) and antibiotic-associated diarrhea (AAD) show specific alterations of the microbial community, called dysbiosis, and these diseases are supposed to be driven at least in part by these alterations [5]–[12]. Nevertheless, it is questionable whether dysbiosis itself causes these diseases or is just an epiphenomenon due to a microbial habitat altered by other pathophysiological factors [11], [12].

A hallmark of many GI diseases is diarrhea, which often correlates with the severity of disease. Diarrhea is characterized by increased stool frequency, decreased stool consistency and increased stool weight. Pathophysiologic mechanisms leading to diarrhea include increased amounts of fluid in the intestinal lumen due to osmotically active substances (osmotic diarrhea), impaired absorption or increased secretion of water and electrolytes (secretory diarrhea) and accelerated intestinal transit [13], [14]. Diarrhea is often caused by a combination of these mechanisms, which furthermore leads to intestinal malabsorption of nutrients such as fat or bile acids, altering the milieu within the gut [15], [16]. Basically, acceleration of the luminal content influences the composition of the microbial community. Microbes that are replicating slowly or experiencing a particle-associated or free-living state will be subjected to wash-out and negatively selected against microbes that adhere to the mucosa or are replicating fast [17]. This principle shows that variation in just one parameter of GI physiology, like increased transit or increased amounts of fluid in the lumen, might have a profound influence on the microbial composition of our gut. Thus, deduction of relevant microbial community alterations in the light of a specific disease must take these accompanying effects into account.

To understand the effects of diarrhea on the composition of the GI microbiota we performed a longitudinal study wherein we induced osmotic diarrhea in four healthy adults by oral administration of polyethylene glycol 4000 (PEG). PEG is a polymer that is not reabsorbed or metabolized by intestinal bacteria. It is a pure osmotic agent that binds water in the intestinal lumen and so leads to diarrhea when administered in higher doses [18]. It is used to treat constipation and to cleanse the bowel prior to endoscopy. Stool as well as mucosa samples were collected before, during and after induction of diarrhea and subjected to culture-independent 16S rDNA-based microbiota profiling using barcoded pyrosequencing.

## Materials and Methods

### Study Protocol

**Figure 1:**
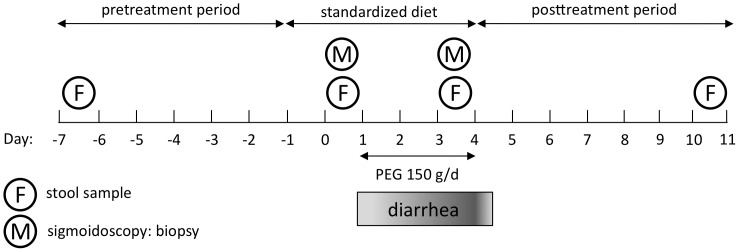
Study design. Subjects were on a free diet from day –7 to day –2 and from day 4 to day 10. From day −1 to day 0 a standardized diet was ingested. Diarrhea was induced by PEG for 3 days (day 1 to day 3). One stool sample was obtained one week before induction of diarrhea. Before the first dose of PEG a second stool sample and a mucosa sample were collected. A third stool and a second mucosa sample were taken at day three of PEG administration when diarrhea was maximally pronounced. A fourth stool sample was taken one week after withdrawal of PEG.

Four healthy adult Caucasian males (subjects A, B, C, D) participated in this study (age range 36–47 years, BMI range 24–26.6). The subjects had had neither antibiotic therapy nor episodes of diarrhea for at least 1 year prior to the study. Stool frequency and consistency were recorded daily during the study and assessed according the Bristol stool chart [19]. After 6 days on a free diet without interventions (pre-treatment period) the subjects were placed on a standard diet (85 g protein, 77 g fat, 250 g carbohydrates, 25 g fiber, total calorie count 2150 kcal/d) for five days. Oral water intake was not restricted. On the third day of the diet diarrhea was induced with the osmotic laxative polyethylene glycol 4000 (Forlax®, Merck, Vienna, Austria) in a dose of 50 g tid (150 g per day). PEG was administered in addition to the standard diet for three days (diarrhea period). Thereafter the subjects again noted their stool behavior without any interventions on a free diet for seven days (post-treatment period) (Fig. 1). The first day of PEG administration and the first day after PEG administration were considered equilibration days and were not included in the analysis of bowel habits. Stool samples were obtained at four different time points. Two baseline samples were taken before induction of diarrhea, sample 1 on a free diet at the beginning of the study (time-point 1, pretreatment period, day −7) and sample 2 seven days later on the second day of the diet (time-point 2, diet period, day 0). Sample 3 was taken from the first stool on the third day of PEG intake while subjects were on the standard diet (time-point 3, diarrhea period, day 3). Sample 4 was taken 7 days after withdrawal of PEG and the standard diet (time-point 4, posttreatment period, day 10). Colonic biopsy samples were obtained from three of the four subjects (subjects B, C, D) at two different time points, sample 1 on the second day of the standard diet before diarrhea was induced (time-point 2, diet period, day 0) and sample 2 on the third day of PEG administration (time-point 3, diarrhea period, day 3). Biopsies were taken from the sigmoid colon 25 cm proximal to the anal canal by flexible sigmoidoscopy without bowel preparation. The mucosa of the area was flushed gently three times with 20 ml of physiological saline solution before two biopsies were taken. Stool samples (abbreviated in figures and tables as F) and mucosa samples (abbreviated in figures and tables as M) were immediately frozen and stored at –20°C.

### Ethics Statement

The study was approved by the institutional review board of the Medical University of Graz (protocol no. 20-090 ex 08/09) and written informed consent was obtained from all subjects.

### DNA Isolation and PCR Amplification

DNA was extracted from stools with the QIAamp DNA Stool Mini kit and from biopsies with the QIAamp DNA Mini Kit (Qiagen, Hilden, Germany) according to the recommended protocol. The stool homogenate was incubated in a boiling water bath for 5 min prior to DNA extraction to increase bacterial DNA yield as recommended. The variable V1–V2 region of the bacterial 16S rRNA gene was amplified with PCR using oligonucleotide primers BSF8 and BSR357 as described previously [20]. This 16S rDNA region was chosen since it gives robust taxonomic classification and has been shown to be suitable for community clustering [21]. We included a sample specific six-nucleotide barcode sequence on primer BSF8 to allow for a simultaneous analysis of multiple samples per pyrosequencing run [22]. Oligonucleotide sequences are given in table S1. PCR conditions were as follows: 100 ng DNA from stool samples or 10 ng from biopsy samples were subjected to PCR amplification in a total volume of 50 µl with 1× HotStar Master Mix (Qiagen) and 20 µM of each primer. For stool samples the following PCR protocol was used: Initial denaturation at 95°C for 12 min followed by 22 cycles of 95°C for 30 sec, 56°C for 30 sec, and 72°C for 1 min and a final step of 72°C for 7 min. For biopsy samples the following PCR protocol was used: Initial denaturation at 95°C for 12 min followed by 35 cycles of 95°C for 30 sec, 56°C for 30 sec, and 72°C for 1 min and a final step of 72°C for 7 min. PCR products were separated on 1% 1xTAE agarose gel and specific bands (∼300 bp) were excised and gel extracted using the Qiagen gel extraction kit (Qiagen). Each sample was amplified and extracted three times independently and subsequently pooled. Purified PCR products were assessed on BioAnalyzer 2100 DNA 1000 chips (Agilent Technologies, Vienna, Austria) for size and integrity. DNA concentration was determined fluorometrically using the QuantiDect reagent (Invitrogen, Carlsbad, CA). An amplicon library was prepared using aequimolar amounts of PCR products derived from the individual samples and bound to the sequencing beads at a one molecule per bead ratio. Long Read Amplicon Sequencing using 70×75 PicoTiter Plates (Roche Diagnostics, Vienna, Austria) was done on a Genome Sequencer FLX system (Roche Diagnostics) according to the manufacturer’s instruction.

### Phylogenetic Analysis

As the initial step the data set was de-noised using the method described by Quince et al. [23], [24] to avoid OTU inflation due to sequencing errors. All sequences shorter than 150 bp containing any ambiguous characters or not matching to the forward primer (distance>2) were discarded [25]. Subsequently, the chimeric sequences were identified with *Uchime* [26] and removed together with contaminant (human) sequences. The remaining sequences were assigned to their respective samples by using the sample-specific 6 bp barcode preceding the primer. In order to perform sample- and time-point-wide comparisons, operational taxonomic units (OTUs) were generated with an extended Ribosomal Database Project (RDP)-Pyrosequencing approach [27], which was integrated in the phylotyping pipeline SnoWMAn (http://SnoWMAn.genome.tugraz.at) [28]. Briefly, all sequences were pooled and aligned with Infernal (V1.0) using a 16S rRNA secondary structure based model for accurate position alignment of sequences [29]. The aligned sequences were clustered by complete linkage to form OTUs with sequence distances ranging from 0% to 5%. For each OTU a representative sequence was extracted and a taxonomic classification was assigned to it using the RDP Bayesian classifier 2.0.1 [30]. Finally, the pooled sequences were again separated according to their sample affiliation. Taxonomic classification and biostatistical analyses reported in this paper were performed on the clustering results for 3% distance.

### Statistical Analysis and Visualization

The analyses were conducted using the statistical environment R (V2.12.1) [31]. Species richness was estimated with the Chao1 estimator [32]. The abundance-based coverage estimator (ACE), diversity and evenness were calculated using the R package “*BiodiversityR*” (V1.5) [33]. Sequence abundance in each sample was normalized to the sample with the maximum number of sequences. Normalization factors ranged between 1.06 and 2.69. Additionally, abundance data were log-2 transformed after adding a value uniformly distributed between 0.75 and 1.25 to downweight OTUs with high abundance and to resemble the normal Gaussian distribution more closely. Principal component analysis (PCA) on the normalized, log-2 transformed data was performed with the *prcomp* function of R. OTUs significantly changing between time points were assessed either with *Metastats* using default settings [34] or the R package “*edgeR*” (V2.14.7) using a linear model accounting for the paired nature of the data [35]. To account for multiple comparisons, p-values were adjusted by the method proposed by Benjamini and Hochberg [36]. Adjusted p-values less than 0.05 were considered statistically significant. Changes between time points on the level of taxonomic ranks were investigated using a paired t-test or a ratio paired t-test. The latter tests the ratio of the relative abundances (time-point 3: time point 2) against 1.

**Table 1:** Performance comparisons of methods used for finding changing phylotypes.

	Number of OTUs			
Method	P-value <0.01	P-value <0.05	Association in at least 2 individuals	Association in at least 1 individual
Stool specimens				
*Metastats*	25	72	n.a.	n.a.
*EdgeR*	2	20	n.a.	n.a.
*Viz*	n.a.	n.a.	61	299
Mucosa specimens				
*Metastats*	18	87	n.a.	n.a.
*EdgeR*	28	79	n.a.	n.a.
*Viz*	n.a.	n.a.	64	232

n.a., not applicable.

**Table 2:** Changing taxa due to diarrhea in stool samples.

OTU	Score#	Method*	Mean % time-point 2	±SD	Mean % time-point 3	±SD	Taxonomic classification+
32	3	MEV	0.049	0.030	0.000	0.000	Bacteria1.0Firmicutes0.66Clostridia0.64Clostridiales0.64Ruminococcaceae0.48Faecalibacterium0.44
89	3	MEV	3.635	2.869	0.056	0.097	Bacteria1.0Firmicutes0.98Clostridia0.98Clostridiales0.98Lachnospiraceae0.9Pseudobutyrivibrio0.36
95	3	MEV	0.086	0.055	0.000	0.000	Bacteria1.0Firmicutes0.99Clostridia0.99Clostridiales0.99Lachnospiraceae0.97Dorea0.54
144	3	MEV	0.148	0.163	0.000	0.000	Bacteria1.0Firmicutes0.99Clostridia0.99Clostridiales0.99Lachnospiraceae0.99Lachnospira0.79
194	3	MEV	2.529	1.296	0.080	0.100	Bacteria1.0Bacteroidetes1.0Bacteroidetes1.0Bacteroidales1.0Rikenellaceae1.0Alistipes1.0
246	3	MEV	0.056	0.021	0.001	0.002	Bacteria1.0Firmicutes0.56Clostridia0.52Clostridiales0.33Lachnospiraceae0.16Sporobacterium0.02
338	3	MEV	0.065	0.069	0.000	0.000	Bacteria1.0Firmicutes0.98Clostridia0.98Clostridiales0.98Lachnospiraceae0.98Roseburia0.95
466	3	MEV	0.135	0.131	0.002	0.004	Bacteria1.0Firmicutes0.75Clostridia0.69Clostridiales0.66Ruminococcaceae0.47Ethanoligenens0.37
783	3	MEV	0.074	0.065	0.000	0.000	Bacteria1.0Firmicutes0.84Clostridia0.83Clostridiales0.83Ruminococcaceae0.52Sporobacter0.17
1005	3	MEV	0.217	0.158	0.002	0.004	Bacteria0.99Bacteroidetes0.97Bacteroidetes0.67Bacteroidales0.67Rikenellaceae0.67Alistipes0.67
1171	3	MEV	0.372	0.235	0.007	0.010	Bacteria1.0Proteobacteria0.52Alphaproteobacteria0.3Sphingomonadales0.14Sphingomonadaceae0.14Sphingosinicella0.12
1660	3	MEV	0.068	0.060	0.000	0.000	Bacteria0.99Bacteroidetes0.97Bacteroidetes0.49Bacteroidales0.49Bacteroidaceae0.31Bacteroides0.31
2361	3	MEV	0.118	0.121	0.000	0.000	Bacteria1.0Firmicutes0.74Clostridia0.74Clostridiales0.74Ruminococcaceae0.58Faecalibacterium0.41
1472	2	MV	0.139	0.092	0.034	0.068	Bacteria1.0Firmicutes0.66Clostridia0.64Clostridiales0.64Ruminococcaceae0.42Faecalibacterium0.38
9	2	MV	0.012	0.019	0.210	0.174	Bacteria1.0Firmicutes1.0Clostridia1.0Clostridiales1.0Ruminococcaceae1.0Faecalibacterium1.0
13	2	MV	4.401	3.270	16.617	12.017	Bacteria1.0Bacteroidetes1.0Bacteroidetes0.99Bacteroidales0.99Bacteroidaceae0.99Bacteroides0.99
16	2	ME	0.000	0.000	0.021	0.011	Bacteria1.0Firmicutes1.0Bacilli1.0Lactobacillales1.0Leuconostocaceae0.91Weissella0.9
21	2	MV	0.018	0.007	0.073	0.034	Bacteria1.0Firmicutes0.87Clostridia0.86Clostridiales0.86Lachnospiraceae0.86Roseburia0.78
41	2	MV	0.123	0.110	0.891	0.449	Bacteria1.0Firmicutes1.0Clostridia1.0Clostridiales1.0Ruminococcaceae1.0Faecalibacterium1.0
66	2	MV	0.052	0.027	0.531	0.263	Bacteria1.0Firmicutes0.98Clostridia0.98Clostridiales0.98Lachnospiraceae0.98Roseburia0.38
74	2	MV	0.093	0.118	0.322	0.127	Bacteria1.0Firmicutes0.92Erysipelotrichi0.89Erysipelotrichales0.89Erysipelotrichaceae0.89Coprobacillus0.78
92	2	EV	0.613	1.203	0.000	0.000	Bacteria0.99Firmicutes0.54Clostridia0.49Clostridiales0.48Incertae Sedis XV0.24Aminobacterium0.22
108	2	MV	0.019	0.020	0.113	0.050	Bacteria1.0Firmicutes0.97Clostridia0.97Clostridiales0.96Lachnospiraceae0.9Roseburia0.57
206	2	MV	0.178	0.148	0.773	0.383	Bacteria1.0Firmicutes0.97Clostridia0.97Clostridiales0.97Ruminococcaceae0.97Faecalibacterium0.96
215	2	MV	0.110	0.094	0.595	0.314	Bacteria1.0Firmicutes0.99Clostridia0.99Clostridiales0.99Lachnospiraceae0.94Lachnospiraceae Incertae Sedis0.35
222	2	MV	0.386	0.153	0.024	0.028	Bacteria0.99Bacteroidetes0.95Bacteroidetes0.56Bacteroidales0.56Bacteroidaceae0.38Bacteroides0.38
343	2	MV	0.049	0.049	0.002	0.004	Bacteria1.0Firmicutes0.99Clostridia0.97Clostridiales0.97Ruminococcaceae0.94Ruminococcus0.93
479	2	MV	0.154	0.144	0.004	0.004	Bacteria1.0Bacteroidetes0.97Flavobacteria0.65Flavobacteriales0.65Cryomorphaceae0.27Fluviicola0.22
501	2	MV	0.091	0.105	0.347	0.206	Bacteria0.98Firmicutes0.84Clostridia0.84Clostridiales0.84Lachnospiraceae0.76Syntrophococcus0.64
534	2	MV	0.309	0.499	1.402	0.483	Bacteria1.0Firmicutes0.96Clostridia0.95Clostridiales0.95Lachnospiraceae0.93Lachnobacterium0.29
561	2	MV	0.380	0.205	0.073	0.078	Bacteria1.0Bacteroidetes1.0Bacteroidetes1.0Bacteroidales1.0Rikenellaceae1.0Alistipes1.0
654	2	MV	0.077	0.076	0.004	0.009	Bacteria1.0Bacteroidetes1.0Bacteroidetes1.0Bacteroidales1.0Rikenellaceae1.0Alistipes1.0
1156	2	ME	0.072	0.071	0.000	0.000	Bacteria1.0Firmicutes1.0Clostridia1.0Clostridiales1.0Ruminococcaceae1.0Acetanaerobacterium0.66
1732	2	ME	0.045	0.031	0.000	0.000	Bacteria1.0Firmicutes0.76Clostridia0.72Clostridiales0.71Incertae Sedis XIII0.29Anaerovorax0.29
1861	2	MV	0.412	0.379	0.028	0.044	Bacteria1.0Bacteroidetes1.0Bacteroidetes1.0Bacteroidales1.0Rikenellaceae1.0Alistipes1.0
1959	2	ME	0.019	0.016	0.000	0.000	Bacteria1.0Firmicutes0.95Clostridia0.95Clostridiales0.95Ruminococcaceae0.5Faecalibacterium0.23
1983	2	EV	0.127	0.170	0.000	0.000	Bacteria1.0Firmicutes0.84Clostridia0.83Clostridiales0.83Ruminococcaceae0.72Faecalibacterium0.44
2034	2	EV	0.081	0.097	0.000	0.000	Bacteria1.0Firmicutes0.68Clostridia0.66Clostridiales0.63Ruminococcaceae0.54Faecalibacterium0.38
3402	2	MV	0.071	0.071	0.001	0.002	Bacteria1.0Firmicutes0.61Clostridia0.6Clostridiales0.6Peptococcaceae0.51Peptococcus0.51

# Score 3, found by all 3 methods; score 2, found by 2 out of 3 methods.

* (M) *Metastats*, (E) *edgeR*, (V) scoring & visualization.

+ Taxonomy string according to RDP classification; the number after the taxon name denotes the similarity score.

### Scoring Approach and Visualization of OTUs According to their Change in Abundance

To visualize the change in OTUs’ abundance in relation to diarrhea we used a scoring system in which we assigned each OTU to a respective increasing/decreasing pattern. In this way, we calculated the mean relative abundance between the pre-diarrhea states (time-point 1 and time-point 2) of each OTU. Together with the corresponding relative abundance values for diarrhea (time-point 3) and post-diarrhea (time-point 4), a three point profile (pre-diarrhea – diarrhea – post-diarrhea) of each OTU could be drawn. Only OTUs experiencing an abundance change of at least 0.05% in relation to the respective sample were included. Subsequently, a scoring system was introduced that assigned values of −1 (decreasing abundance value between two states), +1 (increasing abundance value) or 0 (relative abundance change<±0.05%) to the (two) slopes of this profile. The score for the first slope was multiplied by 3 and added to the score of the second slope, yielding a specific overall score for each OTU that related to one of the nine possible profile patterns. For mucosa samples, which were only represented by pre-diarrhea (time-point 2) and diarrhea (time-point 3) states, three 2-point profiles were generated in a similar fashion. Finally, OTUs were assigned to their respective reaction pattern and these associations were visualized with Cytoscape [37].

**Table 3:** Changing taxa due to diarrhea in mucosa samples.

OTU	Score#	Method*	Mean % time-point 2	±SD	Mean % time-point 3	±SD	Taxonomic classification+
1341	3	MEV	0.000	0.000	0.062	0.035	Bacteria1.0Proteobacteria1.0Gammaproteobacteria1.0Pseudomonadales0.99Pseudomonadaceae0.99Pseudomonas0.67
11	2	MV	2.292	1.527	4.509	0.841	Bacteria1.0Firmicutes0.99Bacilli0.99Lactobacillales0.99Streptococcaceae0.98Lactococcus0.9
13	2	EV	7.117	10.403	0.214	0.319	Bacteria1.0Bacteroidetes1.0Bacteroidetes0.99Bacteroidales0.99Bacteroidaceae0.99Bacteroides0.99
25	2	ME	0.034	0.023	0.000	0.000	Bacteria1.0Firmicutes0.98Clostridia0.98Clostridiales0.98Lachnospiraceae0.96Dorea0.26
27	2	EV	1.720	2.409	0.039	0.061	Bacteria1.0Bacteroidetes1.0Bacteroidetes1.0Bacteroidales1.0Bacteroidaceae1.0Bacteroides1.0
42	2	EV	0.347	0.472	0.019	0.033	Bacteria1.0Firmicutes1.0Clostridia1.0Clostridiales1.0Ruminococcaceae1.0Faecalibacterium1.0
44	2	MV	0.187	0.112	1.069	0.706	Bacteria1.0Proteobacteria1.0Gammaproteobacteria1.0Pseudomonadales0.93Moraxellaceae0.75Acinetobacter0.73
48	2	MV	0.169	0.084	0.051	0.058	Bacteria1.0Bacteroidetes1.0Bacteroidetes1.0Bacteroidales1.0Bacteroidaceae1.0Bacteroides1.0
51	2	MV	0.076	0.043	0.147	0.020	Bacteria1.0Firmicutes0.98Bacilli0.98Lactobacillales0.98Leuconostocaceae0.97Weissella0.97
85	2	EV	0.129	0.116	0.000	0.000	Bacteria1.0Bacteroidetes0.97Bacteroidetes0.97Bacteroidales0.97Bacteroidaceae0.95Bacteroides0.95
89	2	EV	0.243	0.258	0.000	0.000	Bacteria1.0Firmicutes0.98Clostridia0.98Clostridiales0.98Lachnospiraceae0.9Pseudobutyrivibrio0.36
94	2	EV	1.489	1.443	0.040	0.069	Bacteria1.0Bacteroidetes0.99Bacteroidetes0.99Bacteroidales0.99Bacteroidaceae0.99Bacteroides0.99
101	2	MV	0.023	0.030	0.090	0.018	Bacteria1.0Proteobacteria1.0Gammaproteobacteria.0Pseudomonadales0.93Moraxellaceae0.9Acinetobacter0.9
115	2	MV	0.857	0.548	1.711	0.161	Bacteria1.0Firmicutes0.99Bacilli0.99Lactobacillales0.99Streptococcaceae0.98Lactococcus0.98
145	2	EV	1.222	2.022	0.004	0.006	Bacteria1.0Bacteroidetes0.97Bacteroidetes0.97Bacteroidales0.97Bacteroidaceae0.97Bacteroides0.97
150	2	EV	0.385	0.348	0.000	0.000	Bacteria1.0Bacteroidetes0.99Bacteroidetes0.98Bacteroidales0.98Bacteroidaceae0.95Bacteroides0.95
158	2	MV	0.131	0.105	0.354	0.168	Bacteria1.0Proteobacteria1.0Betaproteobacteria1.0Burkholderiales1.0Comamonadaceae0.99Acidovorax0.88
177	2	EV	1.876	2.951	0.000	0.000	Bacteria1.0Bacteroidetes1.0Bacteroidetes1.0Bacteroidales1.0Porphyromonadaceae1.0Parabacteroides1.0
194	2	EV	0.225	0.276	0.000	0.000	Bacteria1.0Bacteroidetes1.0Bacteroidetes1.0Bacteroidales1.0Rikenellaceae1.0Alistipes1.0
212	2	MV	0.717	0.490	1.415	0.189	Bacteria1.0Firmicutes1.0Bacilli1.0Lactobacillales1.0Streptococcaceae1.0Streptococcus1.0
243	2	EV	0.101	0.097	0.000	0.000	Bacteria1.0Firmicutes0.7Clostridia0.68Clostridiales0.67Ruminococcaceae0.28Faecalibacterium0.23
284	2	MV	0.025	0.011	0.098	0.016	Bacteria1.0Proteobacteria1.0Gammaproteobacteria0.98Pseudomonadales0.9Moraxellaceae0.66Acinetobacter0.65
596	2	ME	0.000	0.000	0.022	0.019	Bacteria1.0Proteobacteria1.0Epsilonproteobacteria1.0Campylobacterales1.0Campylobacteraceae1.0Arcobacter1.0
666	2	EV	0.090	0.084	0.000	0.000	Bacteria1.0Firmicutes0.97Clostridia0.96Clostridiales0.96Lachnospiraceae0.94Dorea0.35
681	2	MV	0.094	0.044	0.163	0.025	Bacteria1.0Firmicutes0.98Bacilli0.98Lactobacillales0.97Streptococcaceae0.97Lactococcus0.92
693	2	MV	0.052	0.060	0.184	0.020	Bacteria1.0Proteobacteria1.0Epsilonproteobacteria1.0Campylobacterales0.96Campylobacteraceae0.94Sulfurospirillum0.91
791	2	MV	0.027	0.014	0.082	0.031	Bacteria1.0Firmicutes0.98Bacilli0.97Lactobacillales0.97Streptococcaceae0.97Lactococcus0.97
828	2	MV	0.052	0.033	0.120	0.009	Bacteria1.0Firmicutes0.97Bacilli0.96Lactobacillales0.96Streptococcaceae0.94Lactococcus0.94
901	2	ME	0.018	0.012	0.000	0.000	Bacteria1.0Proteobacteria0.95Betaproteobacteria0.86Burkholderiales0.65Oxalobacteraceae0.4Naxibacter0.34
902	2	ME	0.014	0.006	0.000	0.000	Bacteria1.0Proteobacteria1.0Betaproteobacteria1.0Burkholderiales1.0Incertae sedis 50.87Pelomonas0.82
911	2	ME	0.029	0.014	0.000	0.000	Bacteria1.0Bacteroidetes1.0Bacteroidetes1.0Bacteroidales1.0Bacteroidaceae1.0Bacteroides1.0
953	2	MV	0.038	0.023	0.118	0.066	Bacteria1.0Proteobacteria1.0Betaproteobacteria1.0Burkholderiales1.0Comamonadaceae1.0Acidovorax0.82
987	2	ME	0.014	0.010	0.000	0.000	Bacteria1.0Firmicutes1.0Clostridia1.0Clostridiales1.0Ruminococcaceae1.0Faecalibacterium1.0
1057	2	EV	0.064	0.064	0.000	0.000	Bacteria1.0Bacteroidetes0.98Bacteroidetes0.98Bacteroidales0.98Bacteroidaceae0.98Bacteroides0.98
1257	2	EV	0.128	0.163	0.000	0.000	Bacteria1.0Firmicutes0.93Clostridia0.93Clostridiales0.93Ruminococcaceae0.91Subdoligranulum0.61
1391	2	ME	0.006	0.004	0.000	0.000	Bacteria1.0Firmicutes0.99Bacilli0.99Lactobacillales0.99Leuconostocaceae0.93Weissella0.93
1477	2	MV	0.030	0.025	0.080	0.013	Bacteria1.0Firmicutes0.98Bacilli0.97Lactobacillales0.97Leuconostocaceae0.82Weissella0.81
1497	2	MV	0.020	0.019	0.080	0.033	Bacteria1.0Proteobacteria1.0Gammaproteobacteria1.0Pseudomonadales0.9Moraxellaceae0.9Acinetobacter0.9
1690	2	MV	0.034	0.043	0.120	0.057	Bacteria1.0Proteobacteria1.0Gammaproteobacteria1.0Enterobacteriales1.0Enterobacteriaceae1.0Raoultella0.28
1834	2	MV	0.084	0.066	0.247	0.119	Bacteria1.0TM70.53TM7_genera_incertae_sedis0.53
1993	2	MV	0.033	0.038	0.109	0.041	Bacteria1.0Bacteroidetes1.0Flavobacteria1.0Flavobacteriales1.0Flavobacteriaceae1.0Chryseobacterium0.79
2062	2	ME	0.008	0.006	0.000	0.000	Bacteria1.0Bacteroidetes0.97Bacteroidetes0.94Bacteroidales0.94Bacteroidaceae0.93Bacteroides0.93
2156	2	ME	0.022	0.006	0.001	0.002	Bacteria1.0Bacteroidetes0.99Bacteroidetes0.98Bacteroidales0.98Bacteroidaceae0.97Bacteroides0.97
2229	2	MV	0.025	0.021	0.087	0.018	Bacteria1.0Firmicutes0.98Bacilli0.97Lactobacillales0.97Leuconostocaceae0.94Weissella0.94
6041	2	ME	0.008	0.004	0.000	0.000	Bacteria1.0Proteobacteria0.98Betaproteobacteria0.97Burkholderiales0.9Incertae sedis 50.48Leptothrix0.26

# Score 3, found by all 3 methods; score 2, found by 2 out of 3 methods.

* (M) *Metastats*, (E) *edgeR*, (V) scoring & visualization.

+ Taxonomy string according to RDP classification; the number after the taxon name denotes the similarity score.

### Data Availability

Sequence data generated for this work can be accessed via the EBI short read archive (EBI SRA) under the accession number ERP002098.

## Results

### A Highly Individualized Colonic Microbiota with Different Community Structures in Stools and on the Mucosa

After denoising and filtering the data set for chimeras and contaminant (human) sequences, 452,363 high-quality 16S rDNA sequences with an average length of 246 bp (range 230–277 bp) remained, yielding an average of 20,562 sequences per sample (Table. S2). The RDP classifier (80% bootstrap cutoff) assigned 10 phyla, but only 7 phyla were represented by more than 20 sequences. Most sequences were related to the phyla *Bacteroidetes* (52.6%), *Firmicutes* (43.1%), *Proteobacteria* (4%) and *Actinobacteria* (0.2%) [38].

**Figure 2:**
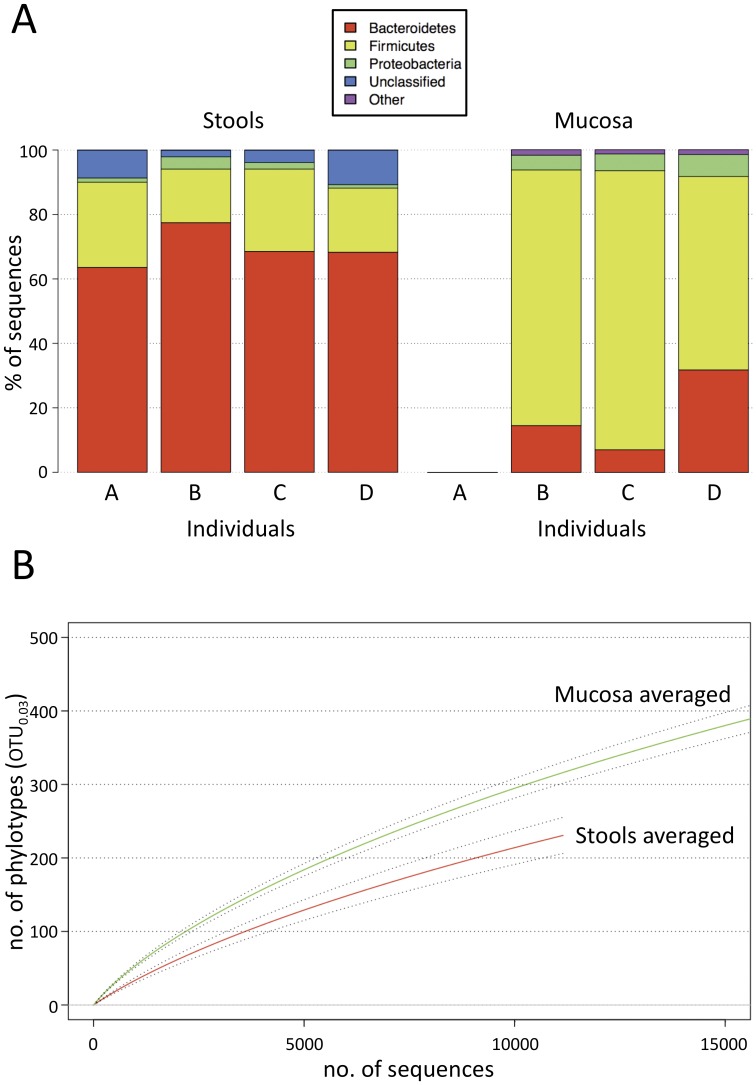
Different community structure and richness in stool and mucosa specimens. (**A**) Relative phylum distribution in stool (individual A, B, C, D) and mucosa specimens (individual B, C, D) from pooled data from each individual. “Unclassified” denotes phylotypes that were only assigned to the bacterial domain by using the 80% identity threshold for RDP classifications. “Other” denotes phyla prevalent below 2%. (**B**) Rarefaction analysis of averaged mucosa (green) and stool (red) samples (OTU distance = 0.03). The dotted line indicates ± SEM.

We noted a strikingly different phylum distribution between stool and mucosa samples. In stools *Bacteroidetes* dominated (69.5±5.8%) followed by *Firmicutes* (22.1±4.7%), whereas on the mucosa *Firmicutes* (75.2±13.7%) were more abundant than *Bacteroidetes* (17.8±12.7%) (Fig. 2A). *Proteobacteria* were also more abundant on the mucosa than in the stools (5.5±11.1% vs. 2.1±1.2%). When the representation of phyla was compared between matched stool and mucosa samples (i.e. from the same individual at the same time point) and p-values were corrected for multiple comparisons, *Firmicutes* (adjusted *P* = 0.001), *Proteobacteria* (adusted *P* = 0.027), *Actinobacteria* (adjusted *P*<0.001) and *Cyanobacteria* (adjusted *P*<0.001) were more abundant on the mucosa and *Bacteriodes* more abundant in stools (adjusted *P* = 0.016). Although we noted a trend towards increased microbial richness on the mucosa compared to stools as indicated by the rarefaction analysis (Fig. 2B), richness was not statistically significant different between the two habitats (*P* = 0.1913 and *P* = 0.989 at time-points 2 and time-points 3, respectively). Microbial diversity and evenness, both measures of the uniformity of the phylotype assembly, also showed no statistical difference between matched stool and mucosa samples (Table. S3).

**Figure 3:**
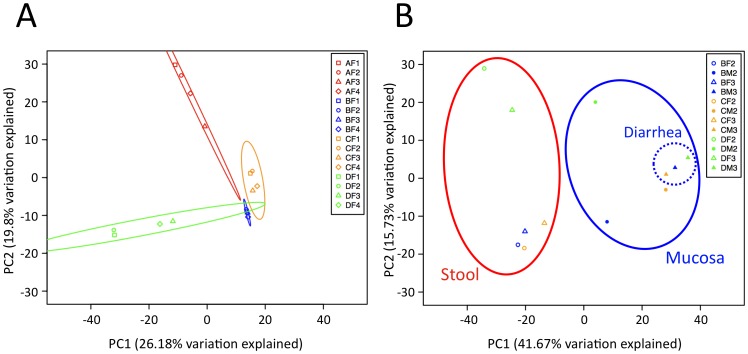
Stool microbiotas are highly individualized and mucosal microbiotas assimilate due to osmotic diarrhea. (**A**) PCA of stool samples according to individuals and treatment periods shows individual specific clustering of stool samples. The principal components 1 & 2 accounting for up to 26.18% variability are shown including 87% confidence ellipses. The inset panels identify the respective samples (A, B, C, D denote subjects; F denotes stool sample; M denotes mucosa sample; time-points: 1, 2 pre-diarrhea, 3 diarrhea, 4 post-diarrhea). (**B**) PCA of stool and the corresponding mucosa samples before and during diarrhea. The principal components 1 & 2 accounting for up to 41.67% variability are shown. Stool and mucosal communities are significantly different (*P* = 0.0002, Student’s t-test) and are clearly separated from each other. Mucosal communities obtained before (time-point 2) and during diarrhea (time-point 3) are significantly different (*P* = 0.0044, Student’s t-test) and cluster independent of the individual, indicating a convergence of the individualized microbiotas.

**Figure 4:**
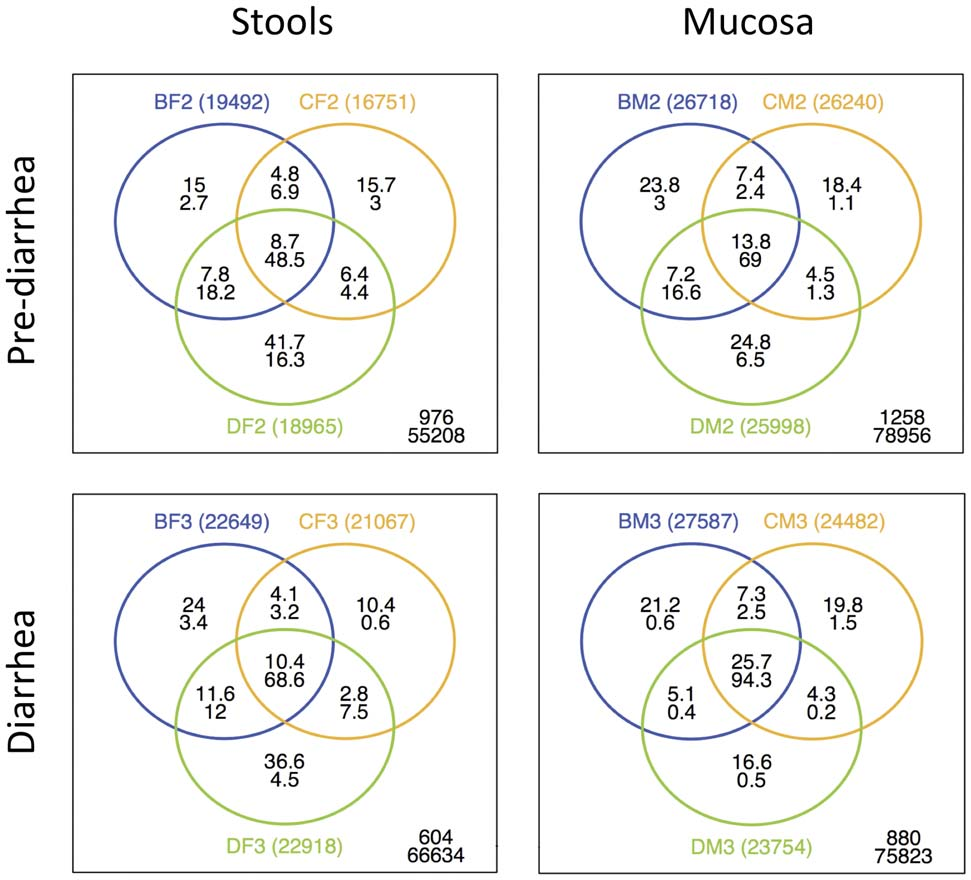
The number of shared phylotypes between individuals increases due to osmotic diarrhea. The number of shared phylotypes (OTU distance = 0.03) between individuals increases during diarrhea in stools (left) but more pronouncedly in mucosa (right) samples (8.7–10.4% vs. 13.8–25.7%). The figure indicates the relative (%) phylotype (upper number) and sequence overlap (lower number) of stool (F) and mucosa (M) samples of individuals B, C and D at time-point 2 (top) and time-point 3 (bottom).

Stool microbiotas were highly individualized; interpersonal variation significantly exceeded intrapersonal variation irrespective of diarrhea (*P*≤0.0077, Student’s t-test) as shown by the principal component analysis (PCA; Fig. 3A). Stool and mucosa samples represented significantly different microbial communities (*P* = 0.0002, Student’s t-test) if matched stool and mucosa samples were analyzed by PCA, which clearly separated the two habitats irrespective of the origin from different individuals (Fig. 3B). Mucosa samples also showed more shared phylotypes between individuals than stool samples and this proportion increased during diarrhea (13.8% vs. 8.7% at time-point 2 and 25.7% vs. 10.4% at time-point 3; Fig. 4).

The most abundant phylotypes across all stool specimens were dominated by *Bacteroidetes*. In three individuals these were represented by *Bacteroides* (individuals B, C and D) resembling the recently published enterotype 1, in one (individual A) by *Prevotella* resembling enterotype 2 [39], [40]. Often the most abundant stool phylotypes were more individual specific and were rarely detected or absent in stool specimens from other individuals (Table. S4). The most abundant phylotypes in mucosa specimens were dominated by lactobacilli (*Weisella, Leuconostoc, Lactococcus*), which were rarely detected or completely absent in stool specimens from the same person, underscoring the difference in microbial habitat composition (Table. S5). Interestingly, the two most abundant mucosal phylotypes matched to the exopolysaccharide producers *Weisella confusa* and *Weisella cibaria* (OTU_61 and OTU_24; BLAST: 100% homology either). Both were also considered stable phylotypes (i.e. no significant relative abundance change in respect to diarrhea; see below).

### Consequences of Osmotic Diarrhea: Reduction of Microbial Richness and Convergence of Individualized Microbiotas on the Mucosa

**Figure 5:**
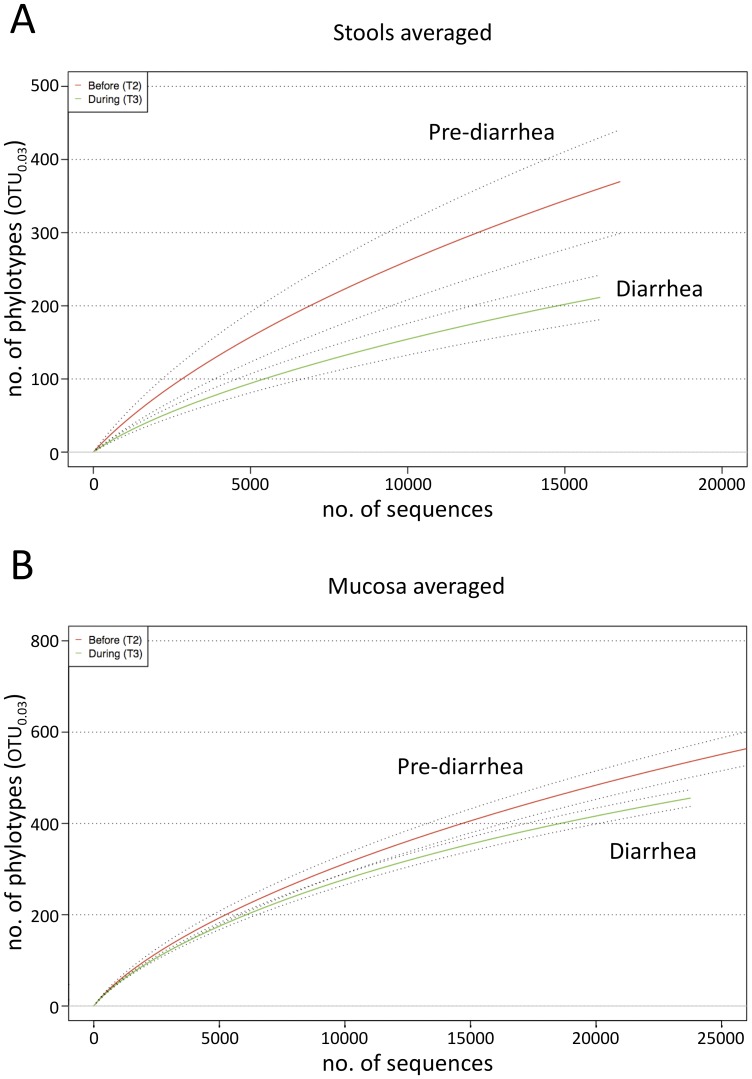
Osmotic diarrhea leads to decreased phylotype richness. (**A**) Rarefaction analysis of averaged stool samples before (time-point 2, red) and during diarrhea (time-point 3, green) shows significantly decreased richness (richness time-point 2 vs. time point 3: *P* = 0.029, Student’s t-test). (**B**) Rarefaction analysis of averaged mucosa samples before (time-point 2, red) and during diarrhea (time-point 3, green) shows a trend toward but non-significant decrease in richness (richness time-point 2 vs. time point 3: *P* = 0.08 Student’s t-test). The dotted line indicates ± SEM; OTU distance = 0.03.

The administration of PEG increased stool frequency (6.0±1.5 vs. 1.2±0.6 bowel movements/day) and decreased stool consistency (stool type: 6.7±0.6 vs. 3.0±0.9) in all 4 individuals (Table. S6). The effect of diarrhea on the individual microbiotas was readily identifiable in the PCA, wherein community variation at time-point 3 exceeded intrapersonal variation between time-points 1 and 2 (Fig. 3). Diarrhea also led to a significant decrease in phylotype richness in stools (*P* = 0.0295, paired t-test), further evidenced by decreased Chao1 and abundance-based coverage (ACE) richness estimators comparing time-point 2 with time-point 3 (*P* = 0.017 and *P* = 0.0218, respectively; Table. S3). Although overall decreased richness due to diarrhea was evident in the rarefaction analysis of mucosa specimens (Fig. 5), this difference did not reach statistical significance (*P* = 0.0801). Phylotype diversity and evenness showed no significant difference between pre-diarrhea and diarrhea samples, either in stools or on the mucosa (Table. S3). PCA clearly separated mucosa from stool samples, reflecting the different niches, and also separated pre-diarrhea mucosa samples by individual. It was noteworthy that diarrhea led to a prominent shift of the mucosal communities, which significantly differed from pre-diarrheal mucosal communities in the PCA (*P* = 0.0044, Student’s t-test). Diarrhea-state mucosal communities clustered together in the PCA, indicating an equalization of the otherwise individualized microbiotas (Fig. 3B). Diarrhea also led to an increase in the number of shared phylotypes between individuals that was most pronounced in the mucosa samples at time-point 3 (Fig. 4).

The capacity of stool microbiotas to reconstitute was assessed by comparing samples from diarrhea (time-point 3) and post-diarrhea (time-point 4). Although species richness increased significantly towards time-point 4 in stools (*P* = 0.042) an overall reduced species richness persisted during the one week interval after PEG administration (Fig. S1; Table. S3).

### Unaltered Community Members in Response to Osmotic Diarrhea

To understand the community changes induced by PEG administration in more detail we assessed the relative abundance change of phylotypes during the course of the study. Depending on the stressor acting on the microbial community (i.e. wash-out due to osmotic diarrhea) and the life-style of the respective microbes (adherent vs. living in suspension), certain phylotypes should experience a more pronounced abundance change compared to others. Thus we assessed the coefficient of variation (CV) of the relative abundances of phylotypes between time-point 2 and time-point 3 samples. A CV of ≤10% was chosen as threshold and only phylotypes prevalent with at least 10 reads per individual were considered. This analysis revealed that only a small fraction of phylotypes exhibited stable behavior and the proportion of these so-called “stable” phylotypes differed greatly between subjects (Table. S7). The majority of stable phylotypes were specific to the individuals, meaning that a phylotype showing stable behavior in one individual showed non-stable behavior in the other individuals according to our definition. In stools only one stable phylotype was found in two individuals simultaneously (OTU_1199; *Lachnospiriaceae*), while there was none in the mucosa samples. In stool samples the stable phylotype with the highest abundance was represented by *Bacteroides vulgatus* (OTU_33; BLAST homology 100%), but only in one individual (Table. S4, Table. S7). In the mucosa samples stable phylotypes with the highest abundance were represented by *Weisella confusa* and *Weisella cibaria* (OTU_61 and OTU_24, respectively; BLAST homology 100%), which also represented top abundant phylotypes on the mucosa as mentioned above (Table. S5). Several low-abundant phylotypes were also considered stable (Table. S7). In general, *Firmicutes* were overrepresented in both mucosa and stool samples as stable phylotypes (Table. S7). The finding that the number of stable phylotypes differed greatly between individuals highlights the high degree of individualization of the GI microbiota. Moreover, stable behavior seems to be related to the individual and/or the microbial community itself and not to the phylotype *per-se*.

### Altered Community Members in Response to Osmotic Diarrhea

We next looked for phylotypes showing a significant relative abundance change in response to diarrhea by comparing time-point 2 with time-point 3 samples. In stools we also assessed significantly changing phylotypes involved in reconstitution by comparing time-point 3 with time-point 4 samples. We initially performed this analysis at the levels of phylogenetic ranks from phylum down to genus. After testing for multiple comparisons only *Rikenellaceae* (family level; adjusted *P* = 0.000), *Alistipes* and *Holdemania* (genus level; adjusted *P* = 0.000 and *P* = 0.032, respectively) showed a significant relative decrease in response to diarrhea in stools (Table. S8). No significantly changing taxon during the reconstitution phase (comparing time-point 3 with time-point 4) could be identified in stools (Table. S9). In the mucosa samples *Rikenellaceae* (family level; adjusted *P* = 0.000) and *Alistipes* (genus level; adjusted *P* = 0.000) also showed a significant relative decrease in response to diarrhea (Table. S10). Interestingly, we noted a relative increase of the proteobacterial taxon *Acinetobacter* (genus level; *P* = 0.038) on the mucosa during diarrhea.

This approach revealed only a few significantly changing taxa. It is now evident that the human GI microbiota is highly individualized [2]. Levels of inter-individual variation might therefore exceed community variation induced by diarrhea. Moreover, our pilot study encompassed a relatively small sample size (n = 22), which hampers stringent statistical assessment. Consequently, both preconditions may have obscured patterns in the microbial community driven by osmotic diarrhea. We thus employed an alternative strategy and assessed abundance changes on the level of individual OTUs with three different measures. Two biostatistical tools well established for assessment of abundance data were employed, *Metastats* and *edgeR*. A not too stringent significance threshold was used in these analyses (*P*<0.05) to account for the relatively small sample size. The third approach involved a scoring system with graphical data visualization (denoted *Viz*), wherein the abundance change of phylotypes (increasing and decreasing in response to diarrhea) was scored and presented within association networks created with *Cytoscape*.

**Figure 6:**
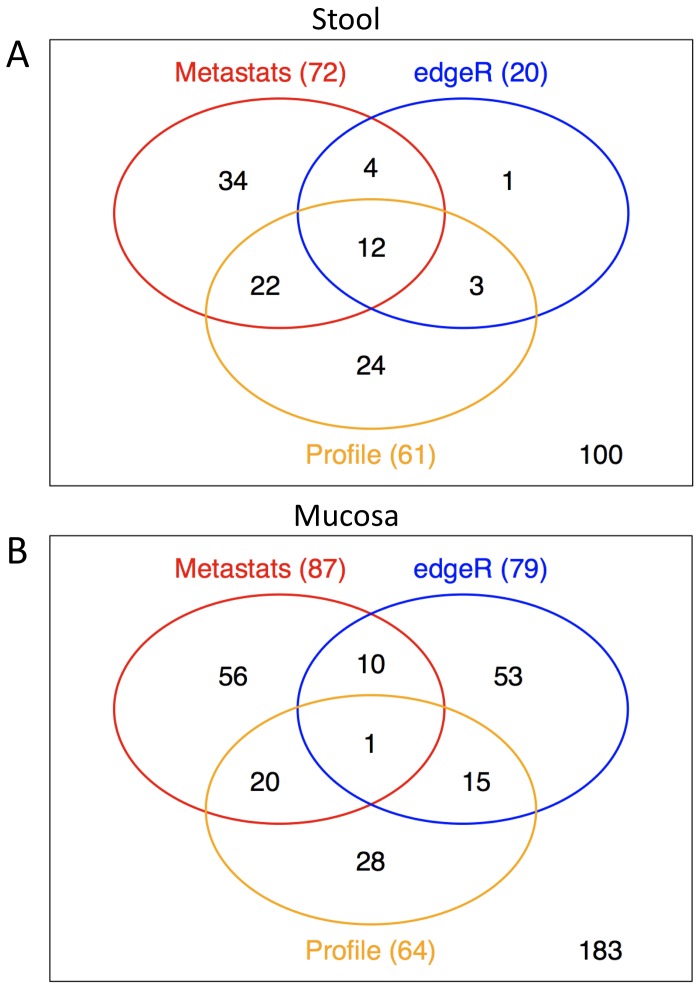
Consistency of measures. Congruence of *Metastats, edgeR* and *Viz* (denoted “Profile”; in at least two individuals simultaneously) for identification of significantly changing OTUs (diversity = 0.03) in stool samples (**A**) and mucosa samples (**B**).

**Figure 7:**
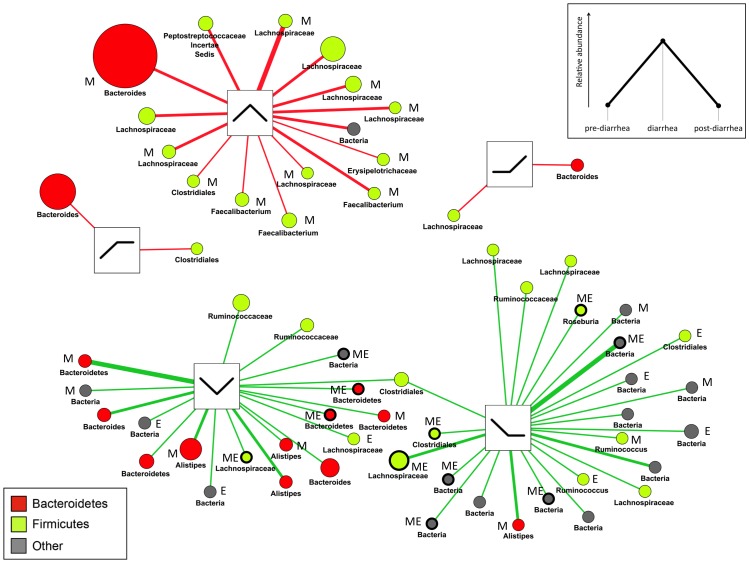
Changing stool OTUs visualized with an association network (*Viz*). OTUs (distance = 0.03) are shown with their respective progression patterns during the study (i.e. abundance change; boxes in the center). The inset exemplifies one possible abundance progression showing an increasing-decreasing pattern. Only OTUs are displayed that were assigned to a respective reaction pattern in at least two individuals (corresponding to thin lines connecting OTUs with their pattern). The width of lines correlates with the number of individuals in whom an OTU was assigned to a specific pattern. Size of nodes correlates with the sum of changes during the study period (mean relative abundance change comparing pre-diarrhea to diarrhea and diarrhea to post-diarrhea samples). OTUs are colored according to their phylum membership and named according to the taxonomic rank conferred by the RDP classifier (80% identity threshold). M denotes significantly changed according to *Metastats* analysis (*P*<0.05); E denotes significantly changed according to *edgeR* analysis (*P*<0.05). OTUs identified by both biostatistical methods are highlighted with a bold outline. Note the increase of *Faecalibacterium* due to diarrhea (upper left) and the skew of *edgeR*-identified phylotypes towards decreasing patterns (bottom).

In stool samples *Metastats* identified 72 significantly changing OTUs and *edgeR* 20 OTUs (Table. S11, S12). *Viz* identified 299 OTUs correlated with a respective reaction pattern (abundance change threshold ≥±0.05%) representing 9.78% of OTUs found in stool specimens. If *Viz* analysis was narrowed down to phylotypes showing a respective association pattern in at least 2 individuals, 61 phylotypes were evident (Table. S13). To that end, all three methods together identified 100 OTUs showing significant relative abundance variation or a respective abundance pattern (in at least 2 individuals) in relation to diarrhea (Table. 1, Fig. 6A) Out of them, 39 OTUs were at least identified by two methods simultaneously (Table. 2). Community variation was readily presented by *Viz*; 37 out of 61 *Viz*-identified phylotypes (60.7%) were reconfirmed by *Metastats* and/or *edgeR* (Fig. 7, Fig. S2). In general, *Bacteroidetes* were associated with an increase and decrease pattern in response to diarrhea but often approached baseline values within the 1 week posttreatment interval. *Firmicutes* were also associated with either an increase pattern and thereafter approached baseline or decreased due to diarrhea and remained so. Interestingly, several OTUs matching to the genus *Faecalibacterium* including *F. prausnitzii* (e.g. OTU_206; BLAST identity 97%) experienced a relative increase in abundance due to diarrhea, which was mirrored by a simultaneous decrease of these taxa in the mucosa specimens.

**Figure 8:**
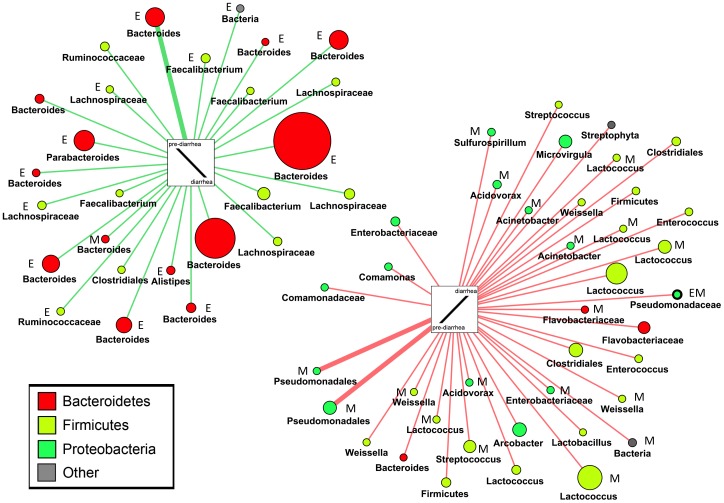
Changing mucosal OTUs visualized with an association network (*Viz)*. OTUs (distance = 0.03) are shown with their respective abundance change comparing pre-diarrhea (time-point 2) with diarrhea (time-point 3) samples. Only OTUs are displayed that were assigned to a respective reaction pattern in at least two individuals (corresponding to thin lines). The width of lines correlates with the number of individuals in whom an OTU was assigned to a specific pattern. Size of nodes correlates with the mean relative abundance change comparing pre-diarrhea to diarrhea samples. OTUs are colored according to their phylum membership and named according to the taxonomic rank conferred by the RDP classifier (80% identity threshold). M denotes significantly changed according to *Metastats* analysis (*P*<0.05); E denotes significantly changed according to *edgeR* analysis (*P*<0.05). OTUs identified by both biostatistical methods are highlighted with a bold outline. Note the increase of various *Proteobacteria,* including opportunistic pathogens (e.g. *Pseudomonas, Acinetobacter, Arcobacter*), and also an increase of *Firmicutes* due to diarrhea (right); *Bacteroidetes* generally occurred together with *Faecalibacterium,* which was mirrored by an increase in stools. Note the skew of *edgeR*-identified OTUs towards the decreasing pattern (left) and of *Metastats* identified OTUs towards the increasing pattern (right).

In the mucosa sample data set, *Metastats* identified 87 significantly changing OTUs and *edgeR* 79 OTUs (Table. S14, S15). *Viz* identified 232 OTUs correlated with a respective reaction pattern (abundance change threshold>±0.05%), representing a fraction of 7.59% of OTUs found in mucosa specimens. If *Viz* analysis was narrowed down to phylotypes showing a respective association pattern in at least in 2 individuals, 64 phylotypes were represented (Table. S16). Given these definitions, all three methods together identified 183 significantly changing OTUs (Table. 1, Fig. 6B). Only one OTU, a *Pseudomonas* sp. (OTU_1341; *Pseudomonas putida*, BLAST identity 100%), was detected by all three methods simultaneously; 46 OTUs were identified at least by two methods simultaneously (Table. 3). Community variation was readily captured by *Viz;* 36 out of 64 *Viz*-identified phylotypes (56.3%) were reconfirmed by *Metastats* and/or *edgeR* (Fig. 8, Fig. S3). Interestingly, several *Proteobacteria* experienced a relative increase in response to diarrhea revealed by *Viz* and confirmed mainly by *Metastats,* as did several lactic acid bacteria. From the 46 OTUs identified by at least 2 methods simultaneously, 13 OTUs (28.3%) represented *Proteobacteria* (Table. 3), among them several opportunistic pathogens including pseudomonads (e.g. OTU_1341, *Pseudomonas putida*, BLAST identity 100%) or the ε-proteobacterial taxon *Arcobacter* (e.g. OTU_596). There was a significant association of *Proteobacteria* with the increasing abundance pattern in *Viz* (*P* = 0.000371, Fisher’s exact test) and a significant association of *Bacteroidetes* with the decreasing pattern (*P* = 0.000216, Fisher’s exact test). As mentioned above several OTUs matching to *Faecalibacterium* including *F. prausnitzii* (e.g. OTU_206) experienced a relative abundance decrease in mucosal specimens (Fig. 8, Table. 3).

## Discussion

We used 16S rDNA-based community profiling to assess the influence of osmotic diarrhea on the composition of the human colonic microbiota. Our longitudinal study with simultaneously sampled stool and mucosa specimens enabled us to compare microbiota changes within and between individuals. We noted strikingly different community structures between stool and mucosa samples wherein *Bacteroidetes* dominated stools and *Firmicutes* the mucosa. The dominance of *Firmicutes* on the mucosa is in accordance with several earlier reports [41], [42]. Bacteria display different life styles: either they are particle associated or they experience a free-living (“planctonic”) life style [17], [43], [44]. Both life styles can be found in stools as well as on the mucosa, although in the latter the polysaccharide-rich mucus overlying the gut epithelium constitutes a biofilm-like community, which might favor a particle-associated life-style [45]. Niche colonization is determined by both partners of the mutualistic human/microbe relationship and is dependent on factors like the availability of nutrients or the capability to adhere [17]. Recent investigations comparing liquid phase and particle-associated communities have also revealed that *Firmicutes* are dominant in the latter [46]. Interestingly, the two top-abundant phylotypes on the mucosa, which have also been found unaltered (“stable”) in response to diarrhea, matched to *Weisella confusa* and *Weisella cibaria* (OTU_61 and OTU_24). Both taxons are exopolysaccharide (dextran) producers and show a strong adhesion capacity, e.g. to Caco-2 cells, which might explain their preferential colonization of the mucosal habitat and their investigation regarding their potential as probiotics [47], [48]. We also recorded a trend toward higher richness on the mucosa compared to stools, which is in accordance with earlier reports [42]. Since the mucosal surface represents the interface of host/microbe interactions, a higher phylotype richness (“biodiversity”), which enhances the robustness and stability of an ecosystem, might be an intrinsic safeguard against perturbations like invasion of pathogens [49], [50]. Understanding the spatial organization of host-associated microbial communities thus poses an important challenge for future microbiota studies of the GI tract [21], [51].

The human GI microbiota shows a high degree of inter-individual variation at higher phylogenetic levels despite a uniform community structure at lower levels where the phyla *Firmicutes* and *Bacteroidetes* dominate [2], [38]. This phenomenon was most prominent in stools, wherein inter-individual differences exceeded any intra-individual variation. In the mucosa samples the degree of inter-individual variation was generally lower, despite a trend towards higher richness. For instance, in mucosa specimens more phylotypes were shared between individuals than in stools. Importantly, diarrhea led to an equalization of the mucosal microbiotas, which clustered together in the PCA and showed an increased phylotype overlap at time-point 3. We induced diarrhea with PEG, a mixture of non-absorbable, non-metabolizable polymers acting as a pure osmotic agent “binding” water in the gut lumen [52]. This led to “wash-out” and decreased phylotype richness in both habitats as described by others [53], [54]. In various inflammatory and diarrheal GI diseases, reduced phylotype richness has been reported, including AAD, *C. difficile* colitis, viral enterocolitis, IBD and IBS [5], [7]–[10], [20], [55]. Reduced richness can be subverted by (opportunistic) pathogens that colonize niches otherwise occupied by the endogenous microbiota [50]. In that regard antibiotic treatment represents a paradigm condition wherein certain groups of bacteria are specifically depleted [55]. Our study indicates that reduced richness *per se* does not necessarily reflect or lead to pathology but is in turn a consequence of the diarrhea prevalent in many GI diseases.

Microbial communities are complex adaptive systems, in which patterns at higher levels emerge from localized interactions and selection processes acting at lower levels [56]. To understand the basic reaction patterns induced by osmotic diarrhea, we assessed the relative abundance change of individual phylotypes. To account for the high level of inter-individual variation of the GI microbiota with our relatively small sample size, we vigorously tested our data set with different approaches. These measures included two established biostatistical tools (*Metastats* and *edgeR*) and a scoring system with graphical representation of the results (*Viz*). These analyses revealed several significantly changing phylotypes but showed reduced congruence between methods. Interestingly, the majority of phylotypes detected with *Viz* (in at least two individuals simultaneously) were confirmed by at least one biostatistical method showing the usefulness of the scoring method. It is important to note that all three methods identified several low abundant significantly changing taxa (i.e. OTUs with about 10 reads representing just about 0.05% of the whole community, given that about 20,000 reads were generated per sample). But reliable detection of these low abundant taxa is highly dependent on the sampling effort (sequencing depth), which can hardly reach completeness given the large number of microbes (about 10^13^–10^14^) colonizing our gut [38]. Thus some of the identified low-abundant OTUs might represent artifacts because of sampling bias. Removal of these low abundant OTUs (e.g. with ≤10 reads) prior to statistical assessment would be a reasonable strategy that might increase accuracy of analysis but could also lead to loss of relevant information [57]–[61].

To overcome the incongruence of the applied methods, we narrowed the findings down to phylotypes that were detected by at least two different methods simultaneously. In this way, we identified several *Bacteroidetes* and *Firmicutes* experiencing a relative increase or decrease in stools in response to diarrhea. On the mucosa *Bacteroidetes* showed a significant association with decreasing relative abundance. It is noteworthy that we observed a significantly increased fraction of *Proteobacteria* experiencing a rise in relative abundance in the mucosa specimens due to diarrhea. Among them were several opportunistic pathogens including pseudomonads like *Pseudomonas* and *Acinetobacter* (e.g. OTU_1341, OTU_101) as well as the ε-proteobacterial taxon *Arcobacter* (e.g. OTU_596). Several lactic-acid bacteria (e.g. *Lactococcus*) also increased on the mucosa during diarrhea, and may therefore represent interesting candidates for probiotics in the setting of diarrheal disease [62]. Interestingly, we also observed a relative increase in taxa matching to *Faecalibacterium* including *F. prausnitzii* (e.g. OTU_206) in stools, which was mirrored by a simultaneous decrease in the mucosa specimens. This observation warrants further investigation since this anti-inflammatory GI bacterium is reported to be decreased in IBD [63], [64].

The finding that *Proteobacteria* increase in response to diarrhea has been reported in several diarrheal and inflammatory GI diseases including IBD [8], [11], [12], [65]–[68]. *Proteobacteria* are usually considered to be generalists able to colonize various habitats with diverse resources. For example we found that OTU_1341 matching to *Pseudomonas putida* significantly increased due to diarrhea; this pathogen shows genomic adaptation to diverse environments but can also cause severe diseases in humans [69]–[72]. Since diarrhea decreases richness, as was reflected by a significant drop in several *Bacteroidetes* and *Firmicutes* in our study, it is reasonable to speculate that *Proteobacteria* can occupy and repopulate these depleted niches more efficiently. It so seems that diarrhea *per se*, irrespective of its etiology, can select for this special community type with increased *Proteobacteria*. It is therefore important to note that these changes may not be specific for diseases like IBD but may represent an epiphenomenon of the wash-out effect due to diarrhea. Moreover, the efficient colonization capacity of *Proteobacteria* might explain the effectiveness of strains like *E. coli* Nissle 1917 used for the therapy of IBD [73]. It is important to note that we assessed the relative abundances of taxa within samples and their relative abundance changes comparing different samples, which does not necessarily translate into absolute changes of taxa, which would require further assessment of specimens (e.g. by means of qPCR).

Capturing the true microbial representation within a sample by cultivation-independent techniques is hampered by various technical challenges. Specimen handling, DNA extraction, PCR amplification and sequencing altogether are causes of bias [57], [59], [74]–[77]. For instance, we compared stool and biopsy samples, which display considerable differences in their composition requiring individual protocols for efficient cell lysis and DNA release from samples. To account for the “rich” matrix composition of stools, we utilized a recommended boiling step prior to DNA extraction from feces, which was not used for biopsies. Several reports emphasized the influence of DNA extraction methods on the outcome of PCR-based microbial community surveys [74]–[77]. Thus we cannot exclude that the different extraction protocols used in our study influenced our findings. In addition to specimen work-up, template concentration, primer sequences and PCR conditions including PCR cycle numbers also influence the assessed community structure [57], [59], [75], [78]. The different sample types (i.e. stools and biopsies) in our study displayed different loads of 16S-targets requiring sample-type specific adjustment of PCR cycle numbers (22 and 35 cycles for stool and mucosa samples, respectively) to prevent PCR substrate exhaustion and to approach a similar end-point of PCR within the linear range of amplification. Increased PCR cycles are reported to skew diversity measures leading to an underestimation of diversity present in the sample [79]. Since we noted a trend towards an increased richness in the mucosa samples compared to stools, albeit not statistically significant, we speculate that the PCR cycle trade-off in our study might have led to underestimation of richness in the mucosa samples. The challenge to optimize the technological accuracy of human microbiome studies poses a major challenge. Inconsistencies may remain even if up-to-date technology with high accuracy combined with a stringent data analysis as in our study are used [57], [80].

Our longitudinal study has revealed several important findings regarding the human GI microbiota and its response to diarrhea. (I) We found that stools and the mucosa represent strikingly different habitats with a different community structure and a different response to stressors like diarrhea. For this reason, studies investigating changes in the GI microbiota in association with specific diseases need to consider that the fecal microbiota does not readily reflect the mucosal community. (II) The finding that *Proteobacteria* relatively increase in response to diarrhea on the mucosa is suggestive of a basic principle of the community in this niche regardless of the cause of diarrhea. When the mucosa is severely affected as in IBD, nutrients like iron derived from blood are available in excess for these efficient colonizers [81]. In turn these bacteria can utilize these resources, i.e. they have developed siderophore uptake systems for iron capture, and so can experience a growth advantage [12], [67], [82]. This phenomenon might then lead to the persistent community change (dysbiosis) noted in IBD, which in turn perpetuates chronic inflammation due to the pro-inflammatory behavior of these bacteria. (III) Our findings show definite changes of the GI microbiota in response to PEG treatment, which is used for bowel cleansing prior to endoscopy. Studies using colonoscopy samples for microbiota analysis need to bear this in mind. (IV) We have shown the usefulness of small-scale longitudinal clinical studies to find relevant microbial community patterns of variation, if data are assessed stringently. In this regard our newly described scoring approach with visualization (*Viz)* is a valuable tool; since it readily illustrates the reaction of the microbiota as a whole, patterns can be caught visually by the investigator.

In summary, our study is proof of the principle that manipulation of basic functions of the human GI tract enables the detection of relevant microbial community changes and highlights the importance of such studies investigating basic (patho-)physiological effects on the GI microbiota.

## Supporting Information

Figure S1 **Rarefaction analysis of pooled stools samples from all 4 time-points.** A reduced richness is seen during diarrhea (T3) and a sustained reduced diversity is evident one week after diarrhea (T4). The dotted line indicates ± SEM.
(PNG)

Figure S2 **Significantly changing stool phylotypes visualized with an association network.** This supplemental figure corresponds to Fig. 7 in the main text. The respective OUT numbers are indicated.
(PNG)

Figure S3 **Significantly changing mucosa phylotypes visualized with an association network.** This supplemental figure corresponds to Fig. 8 in the main text. The respective OUT numbers are indicated.
(PNG)

Table S1 **Oligonucleotide primers used in this study.**
(DOCX)

Table S2 **Read and OTU numbers.**
(XLSX)

Table S3 **Richness, diversity and evenness.**
(XLS)

Table S4 **Most abundant stool phylotypes.**
(DOCX)

Table S5 **Most abundant mucosal phylotypes.**
(DOCX)

Table S6 **Effect of PEG on stool frequency and stool consistency in study subjects.**
(DOCX)

Table S7 **Stable phylotypes.**
(XLS)

Table S8 **Significantly changing taxa between pre-diarrhea and diarrhea stool samples.**
(DOCX)

Table S9 **Significantly changing taxa between diarrhea and post-diarrhea stool samples.**
(DOCX)

Table S10 **Significantly changing taxa between pre-diarrhea and diarrhea mucosa samples.**
(DOCX)

Table S11 **Significantly changing stool phylotypes identified by Metastats.**
(XLSX)

Table S12 **Significantly changing stool phylotypes identified by edgeR.**
(XLS)

Table S13 **Changing stool phylotypes identified by Viz.**
(XLSX)

Table S14 **Significantly changing mucosal phylotypes identified by Metastats.**
(XLSX)

Table S15 **Significantly changing mucosal phylotypes identified by edgeR.**
(XLS)

Table S16 **Changing mucosal phylotypes identified by Viz.**
(XLSX)
