# Supplementary figures and images for: Alterations in the Colonic Microbiota in Response to Osmotic Diarrhea
Source: PLoS One. 2013 Feb 8;8(2):e55817. doi: 10.1371/journal.pone.0055817 (PMC3568139; doi:10.1371/journal.pone.0055817)

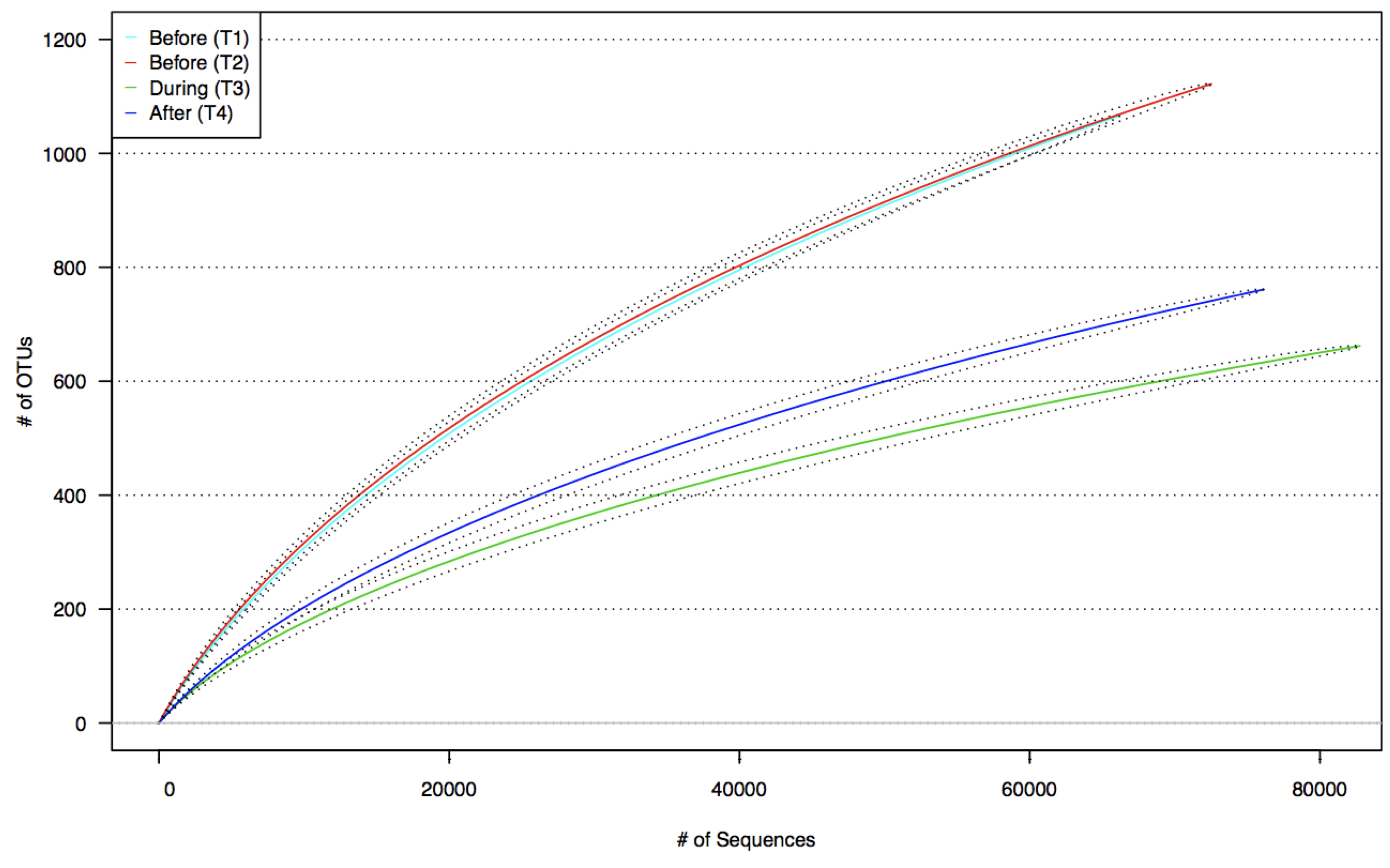

Supplement: Figure S1 — Rarefaction analysis of pooled stools samples from all 4 time-points. A reduced richness is seen during diarrhea (T3) and a sustained reduced diversity is evident one week after diarrhea (T4). The dotted line indicates ± SEM. (PNG) [file pone.0055817.s001.png]

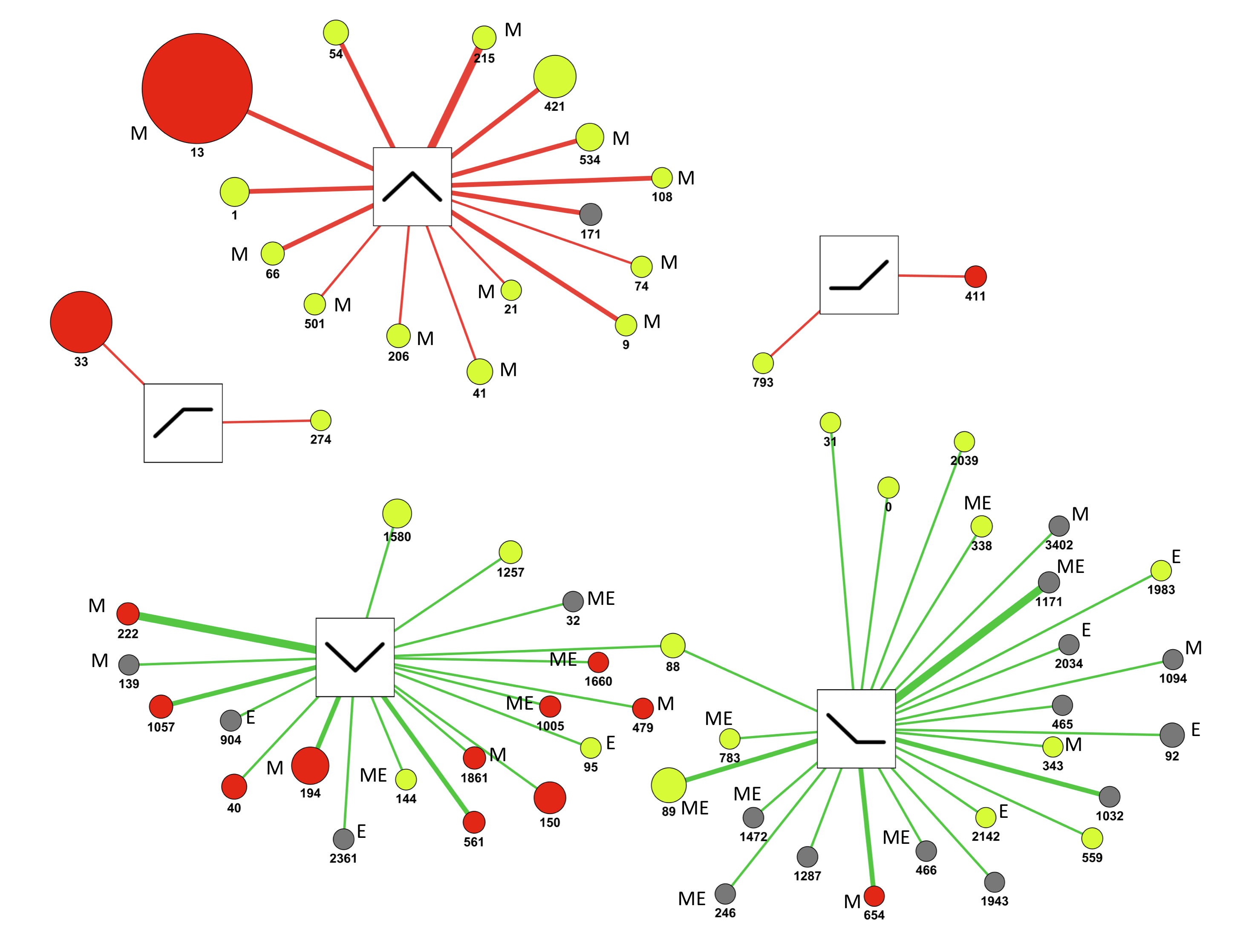

Supplement: Figure S2 — Significantly changing stool phylotypes visualized with an association network. This supplemental figure corresponds to Fig. 7 in the main text. The respective OUT numbers are indicated. (PNG) [file pone.0055817.s002.png]

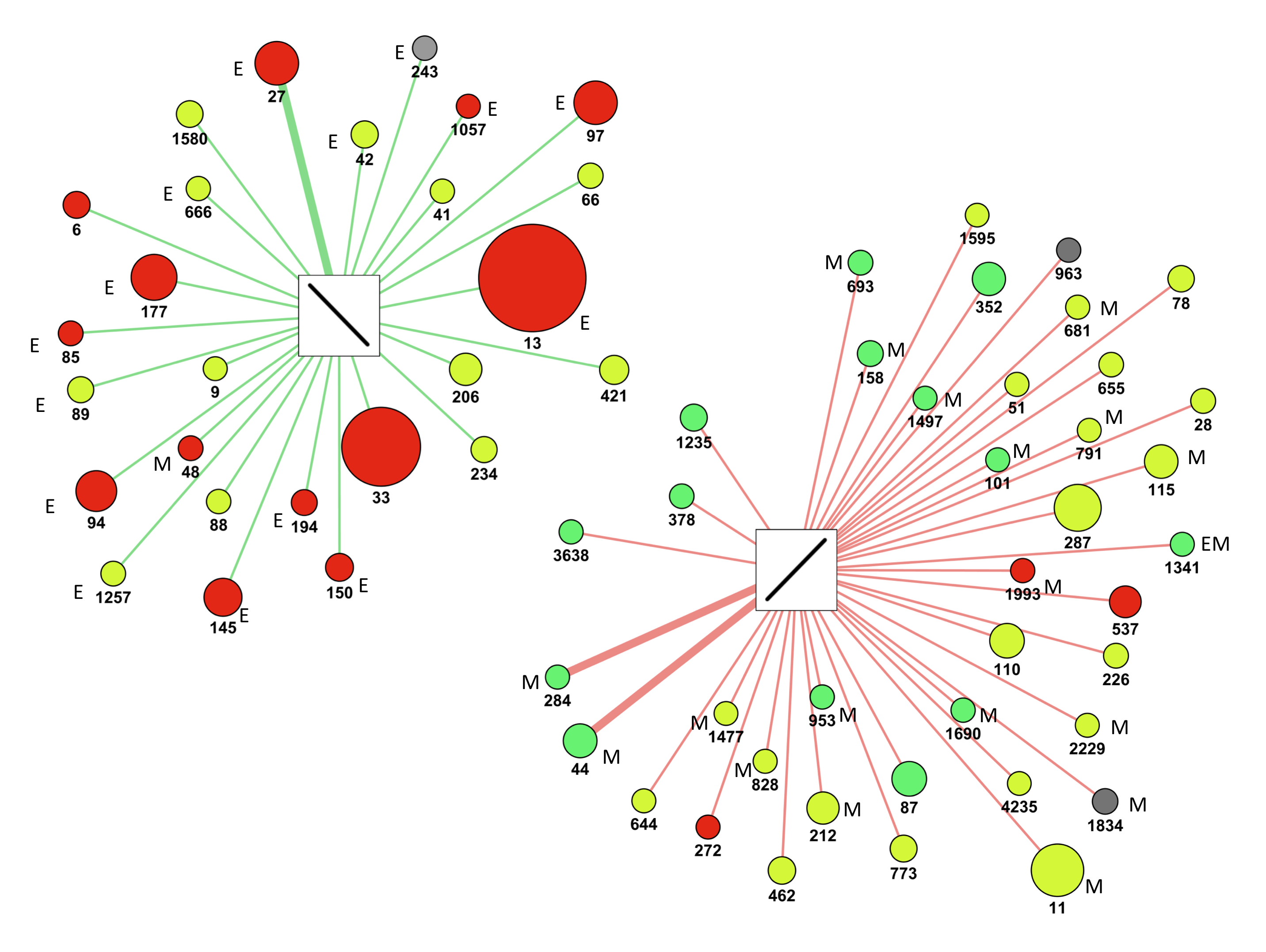

Supplement: Figure S3 — Significantly changing mucosa phylotypes visualized with an association network. This supplemental figure corresponds to Fig. 8 in the main text. The respective OUT numbers are indicated. (PNG) [file pone.0055817.s003.png]
