# Supplementary material for: Alterations in the Colonic Microbiota in Response to Osmotic Diarrhea
Source: PLoS One. 2013 Feb 8;8(2):e55817. doi: 10.1371/journal.pone.0055817 (PMC3568139; doi:10.1371/journal.pone.0055817)
Supplement: Table S1 — Oligonucleotide primers used in this study. (DOCX) [file pone.0055817.s004.docx]

| Table S1. Oligonucleotide primers used in this study | | |
| --- | --- | --- |
| Primer | Sequence 5’ to 3’ | Orientation |
| BSF8_1 | gcctccctcgcgccatcagAGCAGCAGAGTTTGATCCTGGCTCAG | forward |
| BSF8_2 | gcctccctcgcgccatcagCTCAGCAGAGTTTGATCCTGGCTCAG | forward |
| BSF8_3 | gcctccctcgcgccatcagTGCGATAGAGTTTGATCCTGGCTCAG | forward |
| BSF8_4 | gcctccctcgcgccatcagAGATGCAGAGTTTGATCCTGGCTCAG | forward |
| BSF8_5 | gcctccctcgcgccatcagAGCATGAGAGTTTGATCCTGGCTCAG | forward |
| BSF8_6 | gcctccctcgcgccatcagATCATCAGAGTTTGATCCTGGCTCAG | forward |
| BSF8_7 | gcctccctcgcgccatcagATCTGCAGAGTTTGATCCTGGCTCAG | forward |
| BSF8_8 | gcctccctcgcgccatcagATGAGCAGAGTTTGATCCTGGCTCAG | forward |
| BSF8_9 | gcctccctcgcgccatcagATGATGAGAGTTTGATCCTGGCTCAG | forward |
| BSF8_10 | gcctccctcgcgccatcagATGCAGAGAGTTTGATCCTGGCTCAG | forward |
| BSF8_11 | gcctccctcgcgccatcagATGCTCAGAGTTTGATCCTGGCTCAG | forward |
| BSF8_12 | gcctccctcgcgccatcagCAGAGCAGAGTTTGATCCTGGCTCAG | forward |
| BSF8_13 | gcctccctcgcgccatcagCAGATGAGAGTTTGATCCTGGCTCAG | forward |
| BSF8_14 | gcctccctcgcgccatcagCAGCAGAGAGTTTGATCCTGGCTCAG | forward |
| BSF8_15 | gcctccctcgcgccatcagCAGCTCAGAGTTTGATCCTGGCTCAG | forward |
| BSF8_16 | gcctccctcgcgccatcagCATCTGAGAGTTTGATCCTGGCTCAG | forward |
| BSF8_17 | gcctccctcgcgccatcagCATGAGAGAGTTTGATCCTGGCTCAG | forward |
| BSF8_18 | gcctccctcgcgccatcagCTCATGAGAGTTTGATCCTGGCTCAG | forward |
| BSF8_19 | gcctccctcgcgccatcagCTGATCAGAGTTTGATCCTGGCTCAG | forward |
| BSF8_20 | gcctccctcgcgccatcagCTGCTGAGAGTTTGATCCTGGCTCAG | forward |
| BSR357: | gccttgccagcccgctcagCTGCTGCCTYCCGTA | reverse |
