# Supplementary material for: Alterations in the Colonic Microbiota in Response to Osmotic Diarrhea
Source: PLoS One. 2013 Feb 8;8(2):e55817. doi: 10.1371/journal.pone.0055817 (PMC3568139; doi:10.1371/journal.pone.0055817)
Supplement: Table S4 — Most abundant stool phylotypes. (DOCX) [file pone.0055817.s007.docx]

| Table S4. Most abundant stool phylotypes | | | | | | | |
| --- | --- | --- | --- | --- | --- | --- | --- |
| OTU | Number of Reads | Highest relative abundance | Lowest relative abundance | Mean% across all stool specimens | ±SEM | Taxonomic classification^*^ | Comment ^o^ |
| 33 | 28698 | 33.63 | 0 | 10.78 | 2.89 | Bacteria, Bacteroidetes, Bacteroidetes, Bacteroidales, Bacteroidaceae, Bacteroides | Stable phylotype in stools during diarrhea |
| 6 | 27500 | 49.09 | 0 | 9.7 | 4.25 | Bacteria, Bacteroidetes, Bacteroidetes, Bacteroidales, Bacteroidaceae, Bacteroides |  |
| 68 | 19494 | 69.64 | 0 | 8.19 | 4.75 | Bacteria, Bacteroidetes, Bacteroidetes, (Bacteroidales, Prevotellaceae, Prevotella) |  |
| 13 | 21761 | 37.99 | 0 | 7.95 | 2.6 | Bacteria, Bacteroidetes, Bacteroidetes, Bacteroidales, Bacteroidaceae, Bacteroides |  |
| 94 | 12102 | 16.9 | 0 | 4.4 | 1.51 | Bacteria, Bacteroidetes, Bacteroidetes, Bacteroidales, Bacteroidaceae, Bacteroides |  |
| 145 | 10915 | 17.1 | 0 | 3.76 | 1.53 | Bacteria, Bacteroidetes, Bacteroidetes, Bacteroidales, Bacteroidaceae, Bacteroides |  |
| 48 | 10838 | 22.33 | 0.15 | 3.63 | 1.45 | Bacteria, Bacteroidetes, Bacteroidetes, Bacteroidales, Bacteroidaceae, Bacteroides |  |
| 27 | 10417 | 27.29 | 0.05 | 3.57 | 1.76 | Bacteria, Bacteroidetes, Bacteroidetes, Bacteroidales, Bacteroidaceae, Bacteroides |  |
| 89 | 5632 | 7.67 | 0 | 2.03 | 0.63 | Bacteria, Firmicutes, Clostridia, Clostridiales, Lachnospiraceae, (Pseudobutyrivibrio) | Significantly decreases in stools and at the mucosa during diarrhea; butyrate producer; fatty acid production (conjugated linoleic acid); taxon deacreased during social stress in mouse model correlated to increased Il-6 and MCP-1 levels [1] |
| 194 | 6014 | 5.01 | 0 | 2.02 | 0.43 | Bacteria, Bacteroidetes, Bacteroidetes, Bacteroidales, Rikenellaceae, Alistipes | Significantly decreases in stools and at the mucosa during diarrhea; antimicrobial resistance risk pattern (vancomycin, kanamycin, and colistin) [2, 3] |
| 288 | 4251 | 22.07 | 0 | 1.95 | 1.4 | Bacteria, Firmicutes, Clostridia, Clostridiales, Lachnospiraceae, (Parasporobacterium) |  |
| 421 | 5244 | 10.86 | 0.01 | 1.78 | 0.66 | Bacteria, Firmicutes, Clostridia, Clostridiales, Lachnospiraceae, (Pseudobutyrivibrio) |  |
| 150 | 4665 | 16.29 | 0 | 1.67 | 1.02 | Bacteria, Bacteroidetes, Bacteroidetes, Bacteroidales, Bacteroidaceae, Bacteroides |  |
| 49 | 4841 | 20.98 | 0 | 1.62 | 1.31 | Bacteria, Bacteroidetes, Bacteroidetes, Bacteroidales, Bacteroidaceae, Bacteroides |  |
| 177 | 4341 | 10.31 | 0 | 1.62 | 0.63 | Bacteria, Bacteroidetes, Bacteroidetes, Bacteroidales, Porphyromonadaceae, Parabacteroides |  |
| 151 | 4538 | 9.45 | 0 | 1.49 | 0.62 | Bacteria, Bacteroidetes, Bacteroidetes, Bacteroidales, Bacteroidaceae, Bacteroides |  |
| 60 | 4424 | 13.41 | 0 | 1.3 | 0.87 | Bacteria, Bacteroidetes, Bacteroidetes, Bacteroidales, Bacteroidaceae, Bacteroides |  |
| 1580 | 3033 | 3.25 | 0.02 | 1.04 | 0.21 | Bacteria, Firmicutes, Clostridia, Clostridiales, Ruminococcaceae, (Subdoligranulum) |  |
| 97 | 2677 | 5.85 | 0 | 0.97 | 0.43 | Bacteria, Bacteroidetes, Bacteroidetes, Bacteroidales, Bacteroidaceae, Bacteroides |  |
| 1 | 2973 | 4.11 | 0.07 | 0.96 | 0.25 | Bacteria, Firmicutes, Clostridia, Clostridiales, Lachnospiraceae, (Lachnospira) |  |

^*^ Classifications given in parentheses indicates RDP bootstrap < 80%.

Phylotypes are ordered according to mean%.

^o^ References:

[1] Bailey MT, Dowd SE, Galley JD, Hufnagle AR, Allen RG, Lyte M. Exposure to a social stressor alters the structure of the intestinal microbiota: implications for stressor-induced immunomodulation. Brain Behav Immun. 2011; 25(3):397-407.

[2] Rautio M, Lönnroth M, Saxén H, Nikku R, Väisänen ML, Finegold SM, Jousimies-Somer H. Characteristics of an unusual anaerobic pigmented gram-negative rod isolated from normal and inflamed appendices. Clin Infect Dis. 1997;25 Suppl 2:S107-10.

[3] Rautio M, Eerola E, Väisänen-Tunkelrott ML, Molitoris D, Lawson P, Collins MD, Jousimies-Somer H. Reclassification of Bacteroides putredinis (Weinberg et al., 1937) in a new genus Alistipes gen. nov., as Alistipes putredinis comb. nov., and description of Alistipes finegoldii sp. nov., from human sources. Syst Appl Microbiol. 2003;26(2):182-8.
