# Supplementary material for: Alterations in the Colonic Microbiota in Response to Osmotic Diarrhea
Source: PLoS One. 2013 Feb 8;8(2):e55817. doi: 10.1371/journal.pone.0055817 (PMC3568139; doi:10.1371/journal.pone.0055817)
Supplement: Table S5 — Most abundant mucosal phylotypes. (DOCX) [file pone.0055817.s008.docx]

| Table S5. Most abundant mucosal phylotypes | | | | | | | |
| --- | --- | --- | --- | --- | --- | --- | --- |
| OTU | Number of Reads | Highest relative abundance | Lowest relative abundance | Mean% across all stool specimens | ±SEM | Taxonomic classification^*^ | Comment ^o^ |
| 61 | 57225 | 48.09 | 14.81 | 36.24 | 4.75 | Bacteria, Firmicutes, Bacilli, Lactobacillales, Leuconostocaceae, Weissella | Stable phylotype at the mucosa during diarrhea; closest match to *Weisella confusa*, dextran (exopylysaccharide) producing bacterium, β-galactosidase activity [1, 2] |
| 24 | 14222 | 12.75 | 3.45 | 9 | 1.3 | Bacteria, Firmicutes, Bacilli, Lactobacillales, Leuconostocaceae, Weissella | Stable phylotype at the mucosa during diarrhea; closest match to *Weisella cibaria*, dextran (exopylysaccharide) producing bacterium, β-glucosidase activity [1, 2] |
| 37 | 7695 | 6.83 | 1.69 | 5.13 | 0.73 | Bacteria, Firmicutes, Bacilli, Lactobacillales, Leuconostocaceae, Leuconostoc |  |
| 13 | 5742 | 20.24 | 0 | 3.86 | 3.29 | Bacteria, Bacteroidetes, Bacteroidetes, Bacteroidales, Bacteroidaceae, Bacteroides |  |
| 11 | 5215 | 4.91 | 1.35 | 3.3 | 0.63 | Bacteria, Firmicutes, Bacilli, Lactobacillales, Streptococcaceae, Lactococcus |  |
| 33 | 3903 | 7.88 | 0 | 2.49 | 1.3 | Bacteria, Bacteroidetes, Bacteroidetes, Bacteroidales, Bacteroidaceae, Bacteroides |  |
| 287 | 3534 | 3.41 | 0.91 | 2.29 | 0.45 | Bacteria, Firmicutes, Bacilli, Lactobacillales, Streptococcaceae, Lactococcus |  |
| 130 | 3095 | 2.77 | 0.79 | 1.96 | 0.28 | Bacteria, Firmicutes, Bacilli, Lactobacillales, Leuconostocaceae, Weissella |  |
| 60 | 2370 | 8.86 | 0 | 1.48 | 1.48 | Bacteria, Bacteroidetes, Bacteroidetes, Bacteroidales, Bacteroidaceae, Bacteroides |  |
| 115 | 1973 | 1.76 | 0.42 | 1.25 | 0.23 | Bacteria, Firmicutes, Bacilli, Lactobacillales, Streptococcaceae, Lactococcus |  |
| 2 | 1692 | 1.72 | 0.47 | 1.12 | 0.17 | Bacteria, Firmicutes, Bacilli, Lactobacillales, Leuconostocaceae, Weissella |  |
| 212 | 1643 | 1.81 | 0.38 | 1.08 | 0.21 | Bacteria, Firmicutes, Bacilli, Lactobacillales, Streptococcaceae, Streptococcus |  |
| 177 | 1466 | 5.78 | 0 | 1.03 | 0.95 | Bacteria, Bacteroidetes, Bacteroidetes, Bacteroidales, Porphyromonadaceae, Parabacteroides |  |
| 16 | 1471 | 1.14 | 0.4 | 0.93 | 0.11 | Bacteria, Firmicutes, Bacilli, Lactobacillales, Leuconostocaceae, Weissella |  |
| 97 | 1305 | 5.31 | 0 | 0.91 | 0.88 | Bacteria, Bacteroidetes, Bacteroidetes, Bacteroidales, Bacteroidaceae, Bacteroides |  |
| 27 | 1407 | 4.36 | 0 | 0.87 | 0.7 | Bacteria, Bacteroidetes, Bacteroidetes, Bacteroidales, Bacteroidaceae, Bacteroides |  |
| 6 | 1299 | 2.29 | 0 | 0.85 | 0.4 | Bacteria, Bacteroidetes, Bacteroidetes, Bacteroidales, Bacteroidaceae, Bacteroides |  |
| 110 | 1248 | 1.59 | 0.21 | 0.82 | 0.23 | Bacteria, Firmicutes, Clostridia, Clostridiales, (Veillonellaceae, Veillonella) |  |
| 94 | 1215 | 2.79 | 0 | 0.77 | 0.49 | Bacteria, Bacteroidetes, Bacteroidetes, Bacteroidales, Bacteroidaceae, Bacteroides |  |
| 87 | 1161 | 2.16 | 0.17 | 0.74 | 0.3 | Bacteria, Proteobacteria, Epsilonproteobacteria, Campylobacterales, Campylobacteraceae, Arcobacter | Matches to *Arcobacter butzleri* (BLAST: 97% homology); commensal *Campylobacteraceae*? |

^*^ Classifications given in parentheses indicates RDP bootstrap < 80%.

Phylotypes are ordered according to mean%.

^o^ References:

[1] Lee KW, Park JY, Jeong HR, Heo HJ, Han NS, Kim JH. Probiotic properties of Weissella strains isolated from human faeces. Anaerobe. 2012;18(1):96-102.

[2] Bounaix MS, Robert H, Gabriel V, Morel S, Remaud-Siméon M, Gabriel B, Fontagné-Faucher C. Characterization of dextran-producing Weissella strains isolated from sourdoughs and evidence of constitutive dextransucrase expression. FEMS Microbiol Lett. 2010;311(1):18-26.
