# Supplementary material for: Alterations in the Colonic Microbiota in Response to Osmotic Diarrhea
Source: PLoS One. 2013 Feb 8;8(2):e55817. doi: 10.1371/journal.pone.0055817 (PMC3568139; doi:10.1371/journal.pone.0055817)
Supplement: Table S6 — Effect of PEG on stool frequency and stool consistency in study subjects. (DOCX) [file pone.0055817.s009.docx]

| Table S6. Effect of PEG on stool frequency and stool consistency in study subjects (mean ± SD). | | | |
| --- | --- | --- | --- |
|  | Pre-  treatment  (days-7 to 0) | Osmotic diarrhea  (days 2 & 3) | Post-treatment  (days 5 to 10) |
| Stool frequency | 1.2±0.6  (range 0-3) | 6.0±1.5  (range 4-8) | 1.3±0.6  (range 0-2) |
| Stool consistency^a^ | 3.0±0.9  (range 2-5) | 6.7±0.6  (range 6-7) | 3.0±1.1  (range 2-6) |

^a^ according to the Bristol stool chart; range from 1 (hard) to 7 (liquid).
