# Supplementary material for: Alterations in the Colonic Microbiota in Response to Osmotic Diarrhea
Source: PLoS One. 2013 Feb 8;8(2):e55817. doi: 10.1371/journal.pone.0055817 (PMC3568139; doi:10.1371/journal.pone.0055817)
Supplement: Table S8 — Significantly changing taxa between pre-diarrhea and diarrhea stool samples. (DOCX) [file pone.0055817.s011.docx]

| Table S8. Significantly changing taxa between pre-diarrhea and diarrhea stool samples. | | | | | | |
| --- | --- | --- | --- | --- | --- | --- |
| Taxon | Abundance time-point 2 (%) | Abundance time-point 3 (%) | Ratio p-value^*^ | Adjusted ratio p-value | P-value^#^ | Adjusted p-value |
| Class |  |  |  |  |  |  |
| Erysipelotrichi | 0.146±0.147 | 0.442±0.098 | 0.246 | 0.369 | 0.029 | 0.173 |
| Bacilli | 0.007±0.008 | 0.065±0.037 | n.a. | n.a. | 0.05 | 0.173 |
| Order |  |  |  |  |  |  |
| Erysipelotrichales | 0.146±0.147 | 0.442±0.098 | 0.246 | 0.369 | 0.029 | 0.201 |
| Lactobacillales | 0.007±0.008 | 0.065±0.037 | n.a. | n.a. | 0.05 | 0.201 |
| Family |  |  |  |  |  |  |
| Rikenellaceae | 3.6±1.922 | 0.208±0.241 | 0.000 | 0.000 | 0.029 | 0.155 |
| Veillonellaceae | 1.094±0.798 | 0.529±0.737 | 0.029 | 0.086 | 0.091 | 0.226 |
| Erysipelotrichaceae | 0.146±0.147 | 0.442±0.098 | 0.246 | 0.443 | 0.029 | 0.155 |
| Leuconostocaceae | 0.001±0.003 | 0.041±0.02 | n.a. | n.a. | 0.031 | 0.155 |
| Genus |  |  |  |  |  |  |
| Alistipes | 3.6±1.922 | 0.208±0.241 | 0.000 | 0.000 | 0.029 | 0.259 |
| Dialister | 1.091±0.803 | 0.488±0.757 | 0.029 | 0.073 | 0.094 | 0.282 |
| Subdoligranulum | 0.251±0.232 | 0.075±0.092 | 0.022 | 0.073 | 0.121 | 0.289 |
| Holdemania | 0.017±0.007 | 0.062±0.026 | 0.006 | 0.032 | 0.024 | 0.259 |

^*^ Ratio paired t-test (abundance time-point 3/abundance time-point 2 compared to 1).

^#^ paired t-test (abundance time-point 2 compared to abundance time-point 3).
