# Supplementary material for: Alterations in the Colonic Microbiota in Response to Osmotic Diarrhea
Source: PLoS One. 2013 Feb 8;8(2):e55817. doi: 10.1371/journal.pone.0055817 (PMC3568139; doi:10.1371/journal.pone.0055817)
Supplement: Table S9 — Significantly changing taxa between diarrhea and post-diarrhea stool samples. (DOCX) [file pone.0055817.s012.docx]

| Table S9. Significantly changing taxa between diarrhea and post-diarrhea stool samples. | | | | | | |
| --- | --- | --- | --- | --- | --- | --- |
| Taxon | Abundance time-point 3 (%) | Abundance time-point 4 (%) | Ratio p-value^*^ | Adjusted ratio p-value | P-value^#^ | Adjusted p-value |
| Family |  |  |  |  |  |  |
| Peptostreptococcaceae | 0.841±1.544 | 0.096±0.133 | 0.022 | 0.116 | 0.37 | 0.559 |
| Leuconostocaceae | 0.041±0.02 | 0.013±0.015 | 0.026 | 0.116 | 0.077 | 0.492 |
| Genus |  |  |  |  |  |  |
| Peptostreptococcaceae Incertae Sedis | 0.835±1.539 | 0.093±0.131 | 0.015 | 0.085 | 0.37 | 0.654 |
| Weissella | 0.033±0.022 | 0.01±0.009 | 0.015 | 0.085 | 0.099 | 0.56 |

^*^ Ratio paired t-test (abundance time-point 3/abundance time-point 2 compared to 1).

^#^ paired t-test (abundance time-point 2 compared to abundance time-point 3).
