# Supplementary material for: Alterations in the Colonic Microbiota in Response to Osmotic Diarrhea
Source: PLoS One. 2013 Feb 8;8(2):e55817. doi: 10.1371/journal.pone.0055817 (PMC3568139; doi:10.1371/journal.pone.0055817)
Supplement: Table S10 — Significantly changing taxa between pre-diarrhea and diarrhea mucosa samples. (DOCX) [file pone.0055817.s013.docx]

| Table S10. Significantly changing taxa between pre-diarrhea and diarrhea mucosa samples. | | | | | | |
| --- | --- | --- | --- | --- | --- | --- |
| Taxon | Abundance time-point 2 (%) | Abundance time-point 3 (%) | Ratio p-value^*^ | Adjusted ratio p-value | P-value^#^ | Adjusted p-value |
| Family |  |  |  |  |  |  |
| Rikenellaceae | 0.333±0.319 | 0 | 0.00 | 0.00 | 0.213 | 0.667 |
| Veillonellaceae | 0.264±0.303 | 0.04±0.039 | 0.016 | 0.22 | 0.279 | 0.667 |
| Genus |  |  |  |  |  |  |
| Alistipes | 0.333±0.319 | 0 | 0.00 | 0.00 | 0.213 | 0.547 |
| Acinetobacter | 0.055±0.042 | 0.207±0.047 | 0.193 | 0.48 | 0.038 | 0.547 |

^*^ Ratio paired t-test (abundance time-point 3/abundance time-point 2 compared to 1).

^#^ paired t-test (abundance time-point 2 compared to abundance time-point 3).
